# Supplementary material for: Artificial intelligence approaches to the volumetric quantification of glycogen granules in EM images of human tissue
Source: J Gen Physiol. 2024 Jul 9;156(9):e202413595. doi: 10.1085/jgp.202413595 (PMC11233403; doi:10.1085/jgp.202413595)
Supplement: Data S1 — iincludes a description of categorical classification models, their Python codes, and utilities for pre- and postprocessing. [file JGP_202413595_DataS1.pdf]

Supplement 1

Includes (1) a description of Categorical Classification models, (2) their Python codes and (3) Utilities for pre and post processing.

1. Categorical Classification models for the quantification of glycogen granules.

As stated in the main article, we assembled two categorical classifiers, “Locations” and “Granules”, respectively for the region identification and granule-counting tasks. A key component of the categorical approach, which allowed the practical separation of the tasks, was the division of the original EM images into sub-images. The process is illustrated with Supplement 1 Fig. 1. Every EM image (of 4096 x 4096 pixels, at 0.4 nm per pixel, or recast to that pixel distance) was divided into sub-images of 256 x 256 pixels. As an example, the sub-images derived from the portion of original image within the white frame are expanded in the inset.

The subdivision proved advantageous for both tasks: sub-images are squares of 102.4 nm sides, of area smaller than those of the regions of interest; hence, most of them could be assigned entirely to a region. Also, their volume (~0.0006 μ^3) is such that the number of granules within is generally below 10, which in principle reduces the counting task to a classification in 10 classes (0 to 9 granules). As an example, the sub-image in the red frame is assigned to the

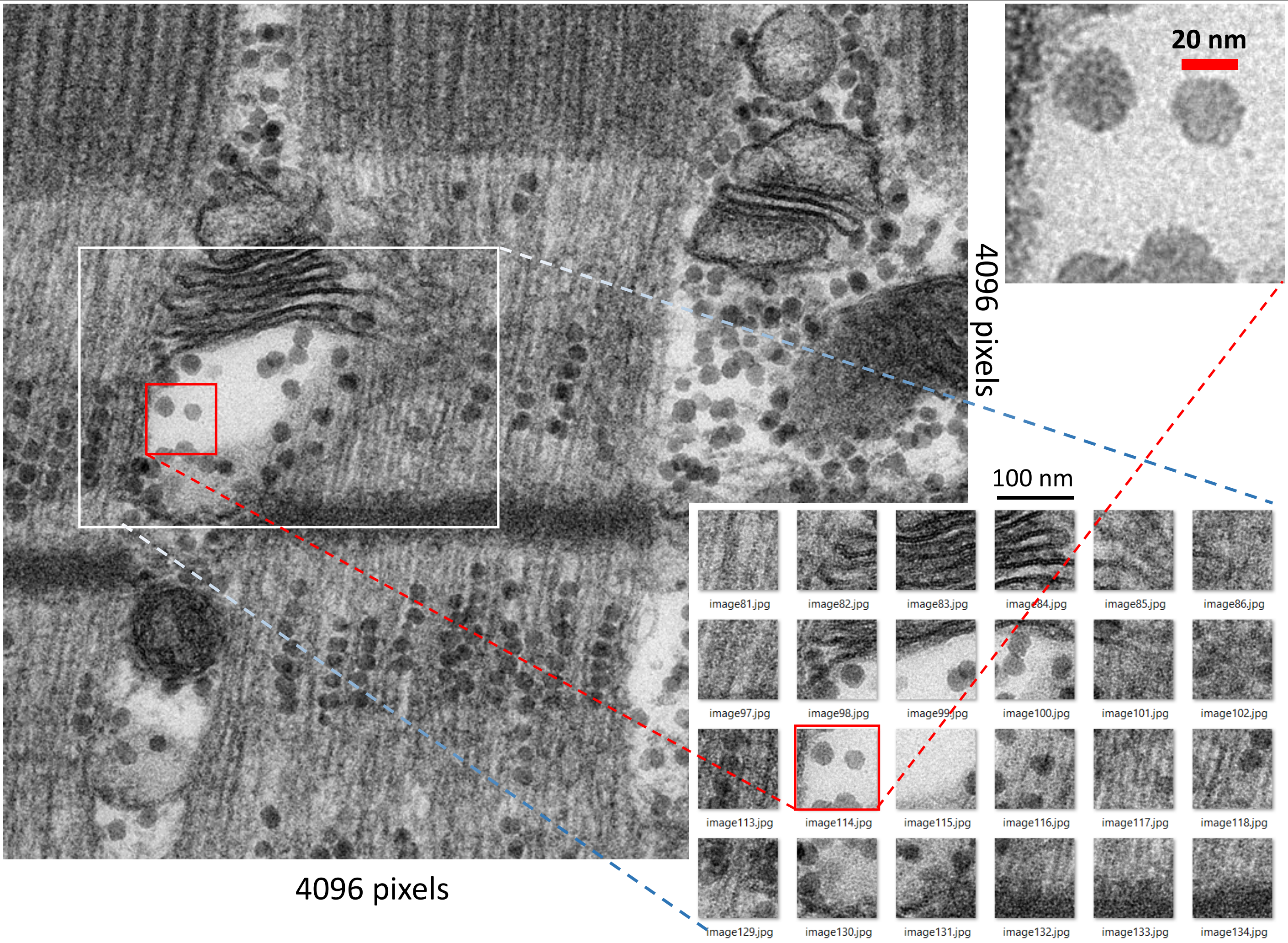

Supplement 1 figure 1. Basis of the categorical classification approach. EM images are split into sub-images. Regardless of original magnification, images are first recast to 0.4 nm/pixel. The images are then split into sub-images of 256 x 256 pixels and 102.4 nm sides. The set at bottom right results from splitting the area within the white frame. Sub-images are submitted to the two AI models for classification according to location and number of granules. As illustrated, sub-images provide reasonable resolution for location within the myofiber, and seldom contain more than 9 granules, thus simplifying the task of the granule counting module. The figure also illustrates that the intermyofibrillar regions can be identified by their content of SR and T tubular membranes, and the absence of filamentous elements.

"3-grain" class by the Granules classifier and to the "near-SR" class (i.e., the inter-myofibrillar cytosol) recognizable for absence of filaments.

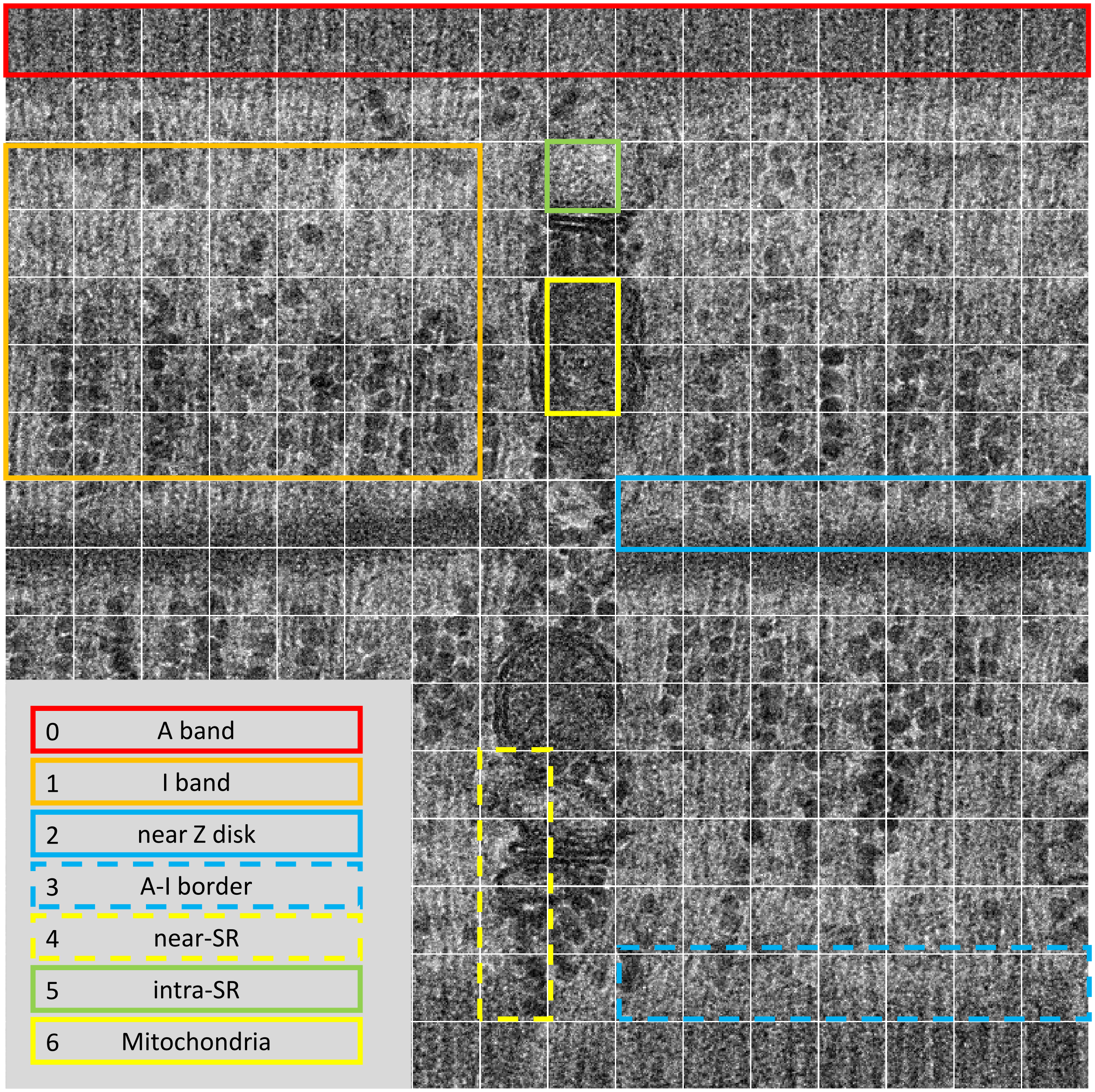

Supplement 1 figure 2. Sub-images are assigned to locations within the myofiber. The "Locations" model is trained to classify sub-images in the 7 classes shown. Each class is represented by a number, from 0 to 6, as listed. Four other classes, numbered 7 to 10 and necessary for technical reasons, are illustrated with figure 4.

1.1 The "Locations" categorical model.

A first model was built to determine the location of every sub-image. 7 location classes of interest were defined: A-band, I-band, Z disk, A-I border, intra-SR, near-SR, mitochondria, illustrated with Supplement 1 Fig. 2. Each of these locations was identified by a digit, as listed in the figure.

The set of 92 EM images obtained with a single microscope at VCU consisted of 62 obtained at 0.77 nm per pixel from 5 human subjects, and 30 at 0.4 nm/pixel. The locations model was applied first to the 15360 sub-images of 256 x 256 pixels derived from 60 of the images in the set at 0.77 nm/pixel (4 human subjects); 512 sub-images, from a 5th patient, became available later. Adding to this group the sub-images derived from the 30 EM images obtained at 0.4 nm/pixel degraded, instead of improving, the overall performance; for this reason, their application was limited to the set acquired at the lower magnification. The segmentation models described in the main article did not have this notable

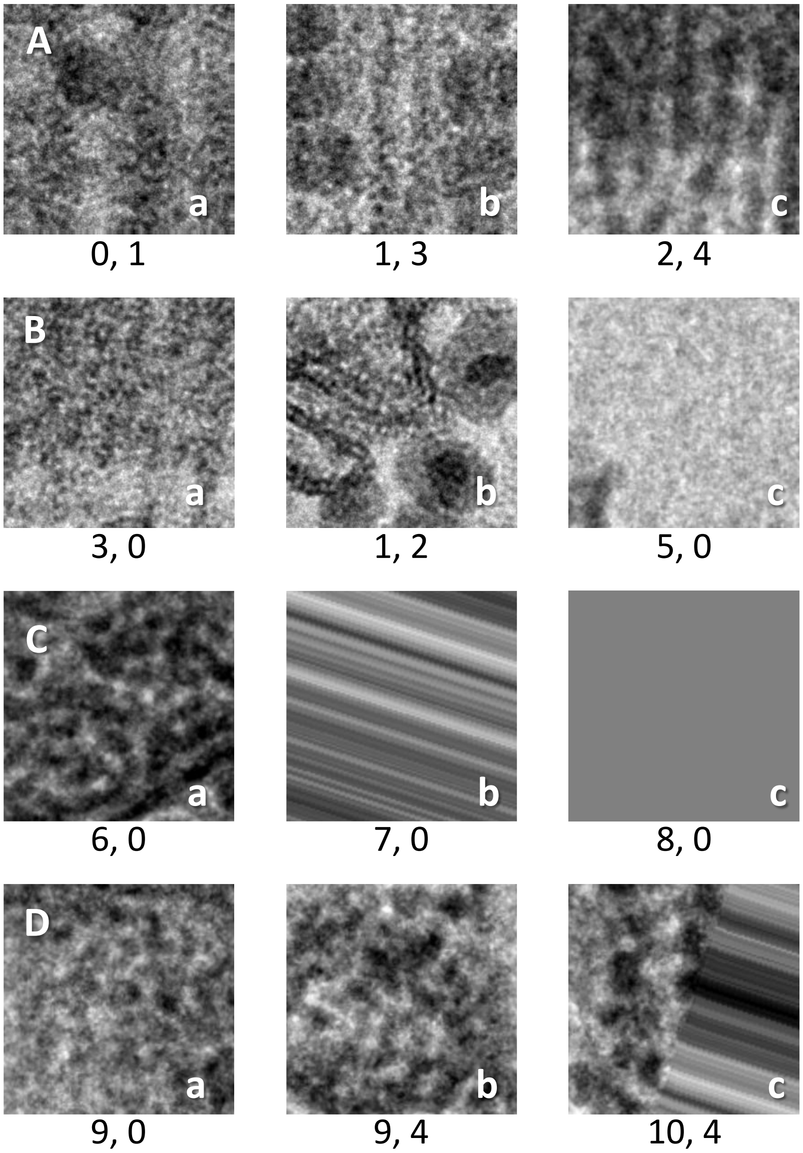

Supplement 1 figure 3. Sub-images in different to locations and granule counts classes. The integers below each sub-image are labels, respectively of location and granules count, assigned by the trainer. Location labels are as listed in Fig. 3, with the addition of '7' and '10' for edge regions of rotated images, '8' for completion edge regions in expanded images, and '9' for sub-images deemed unclassifiable by the trainer. Labels for granule counts are '0' for zero granules, '1' for 1 or 2, '2' for 3 to 5 and '3' for 6 or more granules. '4' was assigned when the number of granules was not clear to the trainer.

limitation.

Both the "Locations" and "Granules" categorical models were built based on "ResNet50" (He et al., 2015), the model that introduced the Residual Networks structure. Code of both models are shared at the end of this Supplement. Our Locations model has 175 layers of units ("neurons"). All layers but the last 3, which carry out the classification task specific to our model, are common to ResNet50, our "base" model.

The 15872 available sub-images (62 x 256) were processed in 4 groups: a training group of 4032 sub-images, a validation group of 1344 sub-images, a test group of 1152, used to quantify performance of the trained model, and the remaining 9344, the "production" set. A single annotator assigned one of the location labels to each sub-image of the training, validation and test sets. The location labels were a total of 11 (0 to 10), as 4 classes had to be added for technical reasons. Sub-images belonging to the 11 location classes are shown in Supplement 1 Fig. 3.

The Locations model has a total of 23,610,000 adjustable parameters, of which 22,539 belong to the layers specific to our problem and the rest are shared with the base model, which is available pre-trained, with parameters optimized to perform image classification on the "Imagenet" database (https://www.image-net.org/). Following transfer-learning practice (Weiss et al., 2016), the optimization or fitting process started from the parameter values (weights) of the base model, and proceeded to gradually increase in successive iterations the numbers of parameters allowed to vary. The maximum accuracy on the validation set (0.819) was obtained by freeing all 7,906,000 parameters between layers # 159 and the final layer, # 275. The Locations model optimized in this way was then tested on 1152 sub-images; 899 of those were labeled correctly, for an accuracy of 0.78. Much of the inaccuracy was due to mis-identifying "technical" regions (borders after rotation, unidentifiable, etc.) which were not included in the final counts, thus not contributing to the overall error.

1.2 The "Granules" categorical model.

It was built using the same procedure described for the Locations model. As explained before, the small sub-images contained a maximum of 9 or 10 granules. Therefore, initially we built an actual "granule counter", namely a classifier model with 11 possible classes or labels (granule counts of 0, 1, …9 or greater; 10 for unclassifiable). This 11-classes ResNet50, was nearly identical to the Locations model. The sole processing difference was the addition of "data augmentation" to the training set, by mirror imaging and rotation of the originals through multiple angles. This operation could not be used for the Locations model, as it would change the

direction angles of the characteristic striations essential for identifying A and I bands. Data augmentation allowed a reduction of the training set to 2560 labeled sub-images.

The initial trials with Granules, however, failed to achieve accuracies better than 0.7. Interpreting this failure as due to lack of accuracy *in the training materials* (i.e., inability of the trainer to assign numbers of granules correctly and consistently), we simplified the task by "binning" counts into 5 classes: class '0' for images with no granules, '1' for 1 or 2 granules, '2' for a 3-5 count, '3' for 6 or more and '4' for the unclassifiable. Thus, the model ceased to be a "counter", becoming a quantifier of lower precision. The Python code and block diagram of the Granules model, which only differs from the Locations model in its last layers, are presented at the end of this Supplement. The validation accuracy of this simplified task reached 0.81 or 81% for a fit that adjusted 5,517,000 parameters, starting at layer 165. On a 1024-image test set, the accuracy reached 0.77. Because the inaccuracies were both in excess and deficit of the "ground truth", the net error in computed totals is likely to be substantially less than the accuracy figure.

		0	1	2	3	4	5	6	7	8	9
		A band	I band	Z disk	A-I border	near SR	intra-SR	mitoch	intra-fibril	inter-fibril	total
1	Sub-images	4805	3542	1382	71	3417	197	823	9800	4437	14237
2	granules	684	1914	121	19	4077	4	44	2738	4125	6863
3	volume, μ^3	3.03	2.22	0.86	0.05	2.15	0.13	0.51	6.16	2.79	8.95
4	concentration, μ^{-3}	226	862	141	380	1896	31	86	444	1478	767

Supplement 1 Table 1. Distribution of glycogen granules derived by categorical classifications. Columns 0 to 6 correspond to classes of the "Locations" model. Column 7 calculates numbers for the entire intra-myofibrillar region by combining entries in columns 0 to 3. Column 4, of class "near-SR", together with column 5 ("Intra-SR"), listing images fully within SR vesicles, and mitochondria, are added together to generate the numbers in the inter-myofibrillar space (Col. 8). Totals, listed in Col. 9, exclude the unclassifiable and completion sub-images. Similar tabulations of the counts from individual subjects, are presented in Supplemental Table 2.

The two categorical classifiers were applied to images from 5 subjects, only one of which was MHN (negative in the MHS diagnostic test). Hence, the results are not suitable for a comparison between MHS and MHN muscles. They do provide information on the distribution per region and are used in the main text for a comparison with the output of the semantic segmenters.

The main quantitative outputs are as follows: the study comprised 8.95 cubic microns of cell space, with an overall concentration of ~767 granules per cubic micron. The near-SR space (or inter-myofibrillar cytosol) contained the highest density of granules, at 1896 per cubic micron. The I band had a ~4 times greater concentration of granules than the A band. There were few granules near mitochondria and the few "intra-SR" ones were likely the result of superposition of intra- and near-SR regions in the 60 nm-thick sections. As described in the main text, the total content of granules evaluated by the semantic segmenters on the same subjects was roughly similar. The regional distribution of granules was also similar. The calculations listed in Table 1 were repeated separately for the images of samples from the 5 individual patients, and are reported in Supplement 1 Table 2. There were differences between subjects, the significance of which cannot be evaluated -- on account of the limited number of patients in this sample. One difference stands out, however; patient #143, the one with the highest Clinical Index (summarizing fairly severe signs and symptoms) had just 233 granules per cubic micron, on account of intra-myofibrillar regions almost devoid of granules as well as a reduced inter-myofibrillar content. A more robust comparison of glycogen content is done in the main text, using the semantic segmentation approach.

1.3 Other Categorical Classifier models.

We built alternative models for the two classification tasks using other available structures. These included VGG-16 (Simonyan and Zisserman, 2015), a Very Deep Convolutional Network model with approximately 138 million parameters that is a current favorite for image identification tasks with large numbers of classes, the Inception model (Szegedy et al., 2015), which with 7 million parameters reached accuracies comparable to VGG-16, and MobileNetV2 (Sandler et al., 2019), which at 3.4 million parameters is designed to be used in small computer systems, including cell phones. The procedures were the same as described for ResNet50, namely, a substitution of the last (or "top") layers for our particular purposes, and use of transfer learning to start from the parameters reached by training the models on the large Imagenet dataset. None of these reached substantially better accuracy in either the locations or granule counting tasks. The only difference noticed was a somewhat lower accuracy of MobileNetV2, but the full exploration of transfer learning that would be needed to affirm that this model is inferior

for the present tasks was not done. In conclusion, with the level of effort that we could muster, we did not find better alternatives to the ResNet50-derived models presented here.

Two-output models. Models that perform simultaneously the assignment of location and the counting of granules were also built. Two-output models have obvious advantages; they simplify and streamline the process, in addition to making possible the immediate computation of variables derived from the two outputs.

To build a simple two-output model we took as input for training a set of images with two labels each (location and number of granules in our case) and provided for two outputs, the corresponding "predictions" of location and granule count. The loss for optimization was calculated jointly from the differences between true value and guesses of location and numbers. As done for the single-output models, the four basic structures (ResNet50, VGG-16, Inception and MobileNet) were tried. The Python listing of the VGG-16 model with dual output is shared at the end of this supplement. Because none reached accuracy of classification comparable to that of the one-output models, the approach was not pursued further.

	A band	I band	Near Z disk	A-I border	Near-SR	SR	Mitoch ondria	Intra-fibrillar	Inter-fibrillar	All regions
**patient 146, MHS**										
Images	1000	1055	114	2	549	56	19	2171	624	2795
Granules	155	205	6	0	981	0	2	366	983	1349
Volume, μ^3	0.63	0.66	0.07	0.00	0.35	0.04	0.01	1.36	0.4	1.76
Concentration, μ^{-3}	246	308	77	0	2841	0	125	269	2458	766
**patient 173, MHS**										
Images	1641	944	277	9	587	1	223	2871	811	3682
Granules	328	944	32	2	1521	0	26	1306	1547	2853
Volume, μ^3	1.03	0.59	0.17	0.01	0.37	0.00	0.14	1.8	0.51	2.31
Concentration, μ^{-3}	317	1589	184	265	4118	0	185	726	3033	1235
**patient 139, MHN**										
Images	1236	1230	436	50	1279	107	188	2952	1574	4526
Granules	168	700	50	17	1226	2	13	935	1241	2176
Volume, μ^3	0.78	0.77	0.27	0.03	0.80	0.07	0.12	1.85	0.99	2.84
Concentration, μ^{-3}	216	905	183	540	1524	22	112	505	1254	766
**patient 143, MHS**										
Images	775	154	501	2	931	32	356	1432	1319	2751
Granules	12	38	33	0	330	2	0	83	332	415
Volume, μ^3	0.49	0.10	0.32	0.00	0.59	0.02	0.22	0.91	0.83	1.74
Concentration, μ^{-3}	24	390	106	0	564	75	0	91	400	239
**patient 145, MHS**										
Images	153	159	54	8	71	1	37	374	109	483
Granules	21	27	0	0	19	0	3	48	22	70
Volume, μ^3	0.10	0.10	0.03	0.01	0.04	0.00	0.02	0.24	0.06	0.30
Concentration, μ^{-3}	213	265	0	0	425	0	129	200	367	233

Supplemental Table 2. Quantity and concentration of glycogen granules, by location and individual patient, as determined by the AI models. Incomplete cells reflect no images placed by the Locations model in the "A-I border" class. The "totals" section of the Table is identical to Table 1 in the article. Note sparsity of granules in patient #143, especially marked in the A and I bands.

2. Python code of the three modules used in the categorical study

2.1. The Locations model in coarse training mode.

Presented as a Jupyter Notebook. Command lines are grouped in cells to mark the basic functions. Notebook functionality allows for individual execution of cells. Cell 1 loads program libraries; 2 and 3 input images and their trainer-assigned labels, using the Dataset format; 4 builds the network structure using Keras's "Functional API" interface. Note that ResNet50, the base model, is imported pre-trained on the Imagenet dataset, and devoid of its "top" (last) layers, so they can be replaced by ones designed for the task at hand. Cell 5 defines the variable "loss", used to quantify and minimize error, as well as the correction or "learning" rate and compiles the program. Cell 6 sets the first stage of training as 10 iterations (epochs) over the training set and starts execution.

1

```
import matplotlib.pyplot as plt
import numpy as np
import os
import tensorflow as tf
import tensorflow.keras.layers as tfl
from tensorflow.keras.preprocessing import image_dataset_from_directory as idd
from tensorflow.keras.layers.experimental.preprocessing import RandomFlip, RandomRotation
from tensorflow.keras.layers import Dense,Flatten
from tensorflow.keras.models import Sequential
from tensorflow.keras.optimizers import Adam
from tensorflow.keras.utils import plot_model
```

2

```
Y = np.loadtxt("C:/Users/erios/images_3_color_15k_labeled/labels_for_locations.txt", dtype='int')
y2list = list(Y)
IMG_SIZE = (224, 224)
```

3

```
directory = "C:/Users/erios/images_3_color_15k_labeled/"
train_dataset = idd(directory, labels=y2list,label_mode='int', shuffle=False,color_mode='rgb',
                batch_size=32,image_size=IMG_SIZE, validation_split=0.25, subset='training',seed=42)
validation_dataset = idd(directory, labels=y2list,label_mode='int', shuffle=False,color_mode='rgb',
                batch_size=32,image_size=IMG_SIZE, validation_split=0.25, subset='validation',seed=42)
preprocess_input = tf.keras.applications.resnet50.preprocess_input
```

4

```
def locations_model(image_shape=IMG_SIZE, data_augmentation=data_augmenter()):
    input_shape = image_shape + (3,)
#   --------------------------------
    base_model = tf.keras.applications.ResNet50(input_shape=input_shape, include_top=False,
                                                weights='imagenet')
#   --------------------------------
    base_model.trainable = False
    print("Number of layers in the base model: ", len(base_model.layers))
    inputs = tf.keras.Input(shape=input_shape)
    #x = data_augmentation(inputs)
    x = preprocess_input(inputs)
    x = base_model(x, training=False)
    x = tfl.GlobalAveragePooling2D()(x)
    x = tfl.Dropout(.2)(x)
    outputs = tfl.Dense(11, activation = "softmax")(x)
    model = tf.keras.Model(inputs, outputs)

    return model
```

5

```
model2 = locations_model(IMG_SIZE, data_augmentation)
model2.compile(optimizer=Adam(learning_rate=0.001),
              loss=tf.keras.losses.SparseCategoricalCrossentropy(from_logits=False),
              metrics=['accuracy'])
```

6

```
initial_epochs = 10
total_epochs = initial_epochs
history = model2.fit(train_dataset, validation_data=validation_dataset, epochs=initial_epochs)
```

2.2. The Granules model illustrated in "prediction" mode.

The structure is nearly identical to that of "Locations". Note in Cell 2 input of images without labels (i.e., granule counts are not given, as they will be predicted by the model). Cell 3 defines the data augmenter. As with the Locations example, Cell 4 implements the construction of the model in coarse tuning form, with the parameters of ResNet50 layers frozen at their pretrained values. Cell 5 defines the fine-tuning conditions, making layers beyond 164 "trainable" and decreasing the learning rate. The last layer launches classification (prediction) of the input images and produces a readable output list.

1

```
import tensorflow as tf
import tensorflow.keras as K
import matplotlib.pyplot as plt
import numpy as np
import PIL
from tensorflow.keras import layers
from tensorflow.keras.layers.experimental.preprocessing import RandomFlip, RandomRotation
from tensorflow.keras.layers import Dense,Flatten
from tensorflow.keras.models import Sequential
from tensorflow.keras.optimizers import Adam
from tensorflow.keras.preprocessing import image_dataset_from_directory as idd
from tensorflow.keras.utils import plot_model
import os
```

2

```
# Use this cell for images subjet to predictions.
BATCH_SIZE = 32
IMG_SIZE = (224,224)
directory = "C:/Users/erios/images_3_color_no_label/"
predict_dataset = idd(directory,labels=None,image_size=IMG_SIZE,batch_size=32,shuffle=False,color_mode='rgb',seed=42)
```

3

```
# Data augmentation is applied before the pretrained model
def data_augmenter():
    data_augmentation = tf.keras.Sequential()
    data_augmentation.add(RandomFlip('horizontal'))
    data_augmentation.add(RandomFlip('vertical'))
    data_augmentation.add(RandomRotation(0.2))
    return data_augmentation
```

4

```
# Model building. Adds ResNet50 model to a Sequential API (ResNet50 is not sequential)
data_augmentation = data_augmenter()
resnet_model = Sequential()
pretrained_model= tf.keras.applications.ResNet50(include_top=False,
        input_shape=(224,224,3), pooling='avg', weights='imagenet')
for layer in pretrained_model.layers:
        layer.trainable=False
resnet_model.add(data_augmentation)  #when using augmentation,
resnet_model.add(pretrained_model)
resnet_model.add(Dense(512, activation='relu'))
resnet_model.add(Dense(5, activation='softmax'))
```

5

```
base_model = resnet_model.layers[1]  # now with fine tuning.
base_model.trainable = True          # Trains more layers and uses a 10x lower learning rate
fine_tune_at = 164
for layer in base_model.layers[:fine_tune_at]:
    layer.trainable = False
resnet_model.compile(loss=tf.keras.losses.SparseCategoricalCrossentropy(from_logits=False),
              optimizer = tf.keras.optimizers.Adam(learning_rate=0.0001), metrics=['accuracy'])
```

6

```
probs = resnet_model.predict(predict_dataset)  # launches execution in "prediction" mode
preds = probs.argmax(axis=-1)
textstring = ("C:/Users/erios/images_3_color_no_label/pred_granules_column.txt")
f = open(textstring, "w")
for i in range(len(preds)):
    f.write("\n"+str(i)+"   "+str(preds[i]))   # produces a two-column text output
f.close()
```

2.3. The essential components of a two-output (Counts and Locations) model.

The example also illustrates the use of a different pretrained model, VGG!6. The base structure must be imported, which is done in Cell 1. Cell 2 builds the model using the "functional API" (a programming interface in the Keras environment, which allows for the structure bifurcation that produces two outputs). The crucial separation of outputs into outputs1 (granule counts, with 5 classes) and outputs2 (locations, with 11 classes) is done in the last lines of model description.

```
from tensorflow.keras.applications.vgg16 import VGG16
```
1

```
# This function implements a model with two outputs, classifying the images for granule numbers and location.
def resnet_model(image_shape=IMG_SIZE, data_augmentation=data_augmenter()):
    IMG_SHAPE = IMG_SIZE + (3,)
    input_shape = IMG_SHAPE    #(224,224,3)
    base_model= tf.keras.applications.VGG16(include_top=False,
                  input_shape=IMG_SHAPE,
                  pooling='avg',
                  weights='imagenet')
    base_model.trainable = False
    inputs = tf.keras.Input(shape=input_shape)
    x = preprocess_input(inputs)
    x = base_model(x, training=False)
    x = tfl.Dense(512, activation='relu')(x)
    outputs1 = tfl.Dense(5, name='granule_count')(x)
    outputs2 = tfl.Dense(11, name='location')(x)

    model = tf.keras.Model(inputs, outputs=[outputs1,outputs2])

    return model
```
2

```
model2 = resnet_model(IMG_SIZE, data_augmentation)    #crucial step.  resnet_model is a function, not a model
```
3

3. Utilities.

Ancillary programs that implement and streamline preprocessing of EM images. All are written in IDL language (Harris Geospatiale, Paris, France). IDL is a “high-level” programming environment with Fortran-like syntax. Programs are commented (green font), for easy transcription to other languages, including Python. Additional guidance in their use will be available upon request to the corresponding author.

3.1. Location_5digit_11_labels.

A program to split and classify EM images into sub-images and labels used by the AI model “Location”.

Produces sub-images in JPEG format and a text file with “labels” 0-10, indicative of location as assigned by the user.

3.2. image_splitter_granule_counter_5digit_5classes.

A similar program, to split images and produce a text file with granule counts assigned by user. Labels output is simplified to 5 classes: 0 for zero granules, 1 for 1 or 2, 2 for 3 to 5, 3 for 6 or greater, 4 for unclassifiable.

3.3. Image_splitter_5digit_no_label.

A program to split EM images into sub-images to be used by either model in “prediction” mode. These sub-images therefore have no labels pre-assigned by the user/trainer. Program will rapidly split all EM images in the input folder, subject to user approval.

3.4. Text manipulation utilities.

Simple programs that modify label files and serve as examples for formatted input/output.

3.4a. correct_label_last_character. A program that allows correction of individual entries in the “labels” text file.

3.4b. add_index_to_labels. Will add an index number to each row in a one-column list of labels.

3.4c. squeeze_labels_to_range. Will reduce the range of labels, for example, from 0-10 to 0-4. Used to reduce the number of classes in Granules model.

3.5. EM_image_filter.

Will apply Lee filter to EM images. Filtering, not used in preprocessing for the AI models, is found to help the visualization for classification by the user/trainer.

3.6. Split_15k_rot.

A program to expand images obtained at 15,000 magnification to an equivalent magnification of 29,000. It also allows for rotation of the original image to align longitudinal striations (myofibrils) “vertically” and then splits the expanded EM image into 4 4096x4096 images suitable for further processing into sub-images.

3.1. Image splitter and labeling for Locations model.

```
pro location_5digit_11_labels
;first part is an image_splitter. Takes one 4096 x 4096 EM image (TIFF) and splits it in 256 (256x256) sub-images
;suitable for analysis by AI programs
;the user enters the location label (0:A  1:I 2:Z 3:A-I,4:SR-AI 5:SR 6:MITO 7:ROTATED EDGES 8:GRAY COMPLETION 9:UNCLASSIFIABLE)
;10 is the label for rotation-limit areas (part rotation, part original image)
;enter ('') means keep same location as last one (important time saver)
;for use with Resnets_11_locations, outputs are in "fake RGB" format, i.e. R, G and B channels have identical content
;labels and images are output to file as soon as they are classified by trainer.
;If program stops or crashes, output text must be closed by the command-line statement close,om. There will be no loss of data.
; EM images are entered and approved for process. After an image is completed (256 sub-images) the user may stop processing
; then, on resumption, the user must answer 'y' to the question whether to append to existing output file.
dummy='' & num=''
inimage=''
side=4096/256                          ; the number of sub-images in either x or y direction (16)
len=side^2                              ; total number of su-images from one EM image (256)
nfirst=3072     ;adjustable starting value for numbering sub-images and labels
k=nfirst
indir='C:\Users\erios\EM_Images\15k_expanded\'        ; input directory
outdir='C:\Users\erios\images_3_color_15k_labeled\here\'  ;folder for sub-image output
outfile=outdir+'labels_for_locations_more_train.txt'    ; text document with labels assigned by trainer
RGBswitch=1
print,'append to existing? answer y or n'           ; implements adding to an existing labels file
read,dummy,prompt = 'append? y or n:'
if dummy eq 'y' then begin
    openu,om,outfile,/get_lun    ;must be opened for write and read ("openu")
    WHILE ~ EOF(om) DO begin
       readf,om,dummy
       k=k+1
    endwhile
    print,'starting output= ',k
;     stop   ;for debugging, use if needed
endif else begin
    openw, om, outfile,/get_lun ;opened for writing only ("openw")

endelse

inlist=file_search(indir,'*.tif')  ;will process every EM image in the folder
n=n_elements(inlist)

y=strarr(n*len+nfirst+100)           ;will contain labels for sub-images, a string array in this version
for m=0 , n-1 do begin   ;will go through folder and offer to skip file
    nfst = k       ;k-nfst then will be the index of sub-images
    infile = inlist(m) & print, 'reading ',infile
    img=read_tiff(infile)
    ;stop ;for debugging
    im_grid=float(img)           ;a grid, showing where image will be split
    for i=257,3841,256 do im_grid(i-2:i+2,*)=255
    for j=257,3841,256 do im_grid(*,j-2:j+2)=255
    imre=congrid(im_grid,768,768,cubic=-0.5)    ;must first reduce size of EM image to 768x768, to show on the screen
    window,0,xsize=768,ysize=768,xpos=0,ypos=0,title=infile     ;defines window for display
    tv,imre,/order                                  ;display
    read,dummy, prompt='enter to continue, "c" change, "s" stop, "e" end & output, '   ;reject image with s
    if dummy eq 'c' then goto, jumpskipimage
    if dummy eq 's' then stop
    if dummy eq 'e' then goto, jumptoout                            ;get out of processing loop
    ; will split image into 256 sub-images
    imarr=bytarr(len,256,256)
    for j=0,side-1 do begin    ;j is row index
        for i=0,side-1 do begin   ;i is column index --horiz. coord.--. rows will be completed first
            p=k-nfst              ;index of sub-images within image m
            imarr(*,*,p)=img(256*i:256*(i+1)-1,256*j:256*(j+1)-1)
            loc='row '+strcompress(string(j),/remove_all)+' col '+strcompress(string(i),/remove_all)
            window,1,xsize=256,ysize=128,xpos=778,ypos=0, title=loc ;for display of individual sub-image
            tv,congrid(imarr(*,*,p),128,128,cubic=-0.5),/order   ;which is shown at half size for visibility
            strk=string(k)
               print,'image ',strk                          ;sub-image index (counter) converted to string
            if k lt 10 then strk='0000'+ strk
            if (k gt 9) and (k lt 100) then strk = '000'+ strk   ;counter must have 5 digits, for sorting
            if (k gt 99) and (k lt 1000) then strk = '00'+ strk
            if (k gt 999) and (k lt 10000) then strk = '0'+ strk
            print,k,'; '+ loc + 'enter region '                ;classification by user done here


            print,'0:A   1:I   2:Z   3:A-I   4:SR-AI   5:SR 6:MIT 7:ROT 8:GRAY 9:?
            read, num,prompt = ''':KEEP 0:A  1:I 2:Z 3:A-I,4:SR-AI 5:SR 6:M 7:ROT 8:GRAY 9:? c-go back: '
            if num eq '' then num= old
            if num eq 'u' then num= '9'                     ;the AI modules require a numeral (0 to 10)
            if num eq 'a' then num= '0'                     ;but the user here may also introduce a letter
            if num eq 'i' then num= '1'                     ; a for A band, i for I band, z for Z disk, etc.
            if num eq 'z' then num= '2'
            if num eq 'r' then num= '10'
            if num eq 'm' then num= '6'
            if num eq 's' then num= '5'
            if num eq 'g' then num= '8'
            if num eq 'ai' then num= '3'
            if num eq 'si' then num= '4'
            if num eq 'rr' then num= '7'
            old= num
            y(k)=num
            print,'file',k,' is in ', y(k),' region'
            imout=intarr(3,256,256)      ;conversion to RGB
            for q=0,2 do imout(q,*,*)=imarr(*,*,p)
            outname=strcompress(outdir+'image'+strk+'.jpg',/remove_all)      ;produces the name of the sub-image on output
            write_jpeg,outname,imout,quality=100,/order,/true ;sub-images written in JPEG format
            printf,om, k, y(k), format='(I8,A8)'       ;labels y(k) are preceded by the image number 'k'; one per line in text fil
            k=k+1
         endfor
    endfor   ;ENDS LOCATION LABELING AND OUTPUT OF SUB-IMAGES OF ONE EM IMAGE
    jumpskipimage:
    ;stop  ;debugging
endfor       ;completed loop over EM images
print,'last output file number: ',strk   ;equal to # of sub-images minus one.
n_sub_images=fix(strk)+1 & print, '# of sub-images: ',n_sub_images
read,dummy, prompt='enter to continue to text output'
if dummy ne '' then stop
jumptoout:                ;normal end from within main loop
close,om & free_lun,om
print,'labels output to "'+ outfile + '"'
stop
end
```

3.2. Image splitter and labeling for granule counter

```
pro image_splitter_granule_counter_5digit_5classes
;similar to the locations program (Utilities 1). See annotations there
;;output is simplified to 5 levels: 0 granules, 1 or 2, 3 to 5, 6 or more, unclassifiable
;the display of the sub-image for counting is compressed to half size
side=4096/256
len=side^2
nfirst=2816                          ; added sub-images starting at 2816
k=nfirst
dummy='' & num=''
outdir='C:\Users\erios\images_for_training_granules_15k\' & sub='images_here\'  ;output folder for sub-images
outfile=outdir+'Y_list2.txt'    ;will contain labels M
print,'append to existing? answer y or n'
read,dummy,prompt = 'append? y or n:'
if dummy eq 'y' then begin
    openu,om,outfile,/get_lun    ;must be opened for write and read
    WHILE ~ EOF(om) DO begin
       readf,om,dummy
       k=k+1
    endwhile
    print,'starting output= ',k + nfirst  ;just keeping track during execution
endif else begin
    openw, om, outfile,/get_lun        ;open for writing (new)
endelse
indir='C:\Users\erios\EM_Images\15k_expanded_filtered\'      ;add 15k expanded files
;indir='C:\Users\Eduardo\EM_Images\'     ;for laptop
inlist=file_search(indir+'*.tif')   ;note, will use all tiff images in folder
                        ;must reject the ones already done
n=n_elements(inlist)
;y=intarr(n*len)          ;will contain labels for sub-images
y=strarr(n*len+100)           ;will contain labels for sub-images, a string array in this version
for m=0 , n-1 do begin   ;will go through directory and offer to skip file
    nfst = k       ;k-nfst then will be the index of sub-images
    infile = inlist(m) & print, 'reading ',infile
    img=read_tiff(infile)
    im_grid=float(img)
    for i=257,3841,256 do im_grid(i-2:i+2,*)=255
    for j=257,3841,256 do im_grid(*,j-2:j+2)=255
    imre=congrid(im_grid,768,768,cubic=-0.5)
    window,0,xsize=768,ysize=768,xpos=0,ypos=0,title=infile
    tv,imre,/order
    read,dummy, prompt='enter to continue, "c" change, "s" stop, "e" end & output, '   ;reject image with s
    if dummy eq 'c' then goto, jumpskipimage
    if dummy eq 's' then stop
    if dummy eq 'e' then goto, jumptoout
    ; will split image into 256 sub-images
    imarr=bytarr(len,256,256)
        for j=0,side-1 do begin    ;j is row index
        for i=0,side-1 do begin   ;i is column index --horiz. coord.--. rows will be completed first
            p=k-nfst           ;index of sub-images within image m
            imarr(*,*,p)=img(256*i:256*(i+1)-1,256*j:256*(j+1)-1)
       loc='row '+strcompress(string(j),/remove_all)+' col '+strcompress(string(i),/remove_all)
            window,1,xsize=256,ysize=128,xpos=778,ypos=0, title=loc
            tv,congrid(imarr(*,*,p),128,128,cubic=-0.5),/order
            strk=string(k)
            if k lt 10 then strk='0000'+ strk
            if (k gt 9) and (k lt 100) then strk = '000'+ strk
            if (k gt 99) and (k lt 1000) then strk = '00'+ strk
            if (k gt 999) and (k lt 10000) then strk = '0'+ strk
            print,k,'; '+ loc +'; enter number of granules, string, "" for 0, "s" stop'
            read, num,prompt = 'enter # granules,0-8,  9 or u = unknown, "s" stop: '
         if num eq 's' then stop   ;
         if num eq '' then begin     ;simplifies entering 0
            y(k) = '0'
            goto, jumpcase
         endif
         if num eq 'u' then begin     ; unknown
            num = '9'
         endif
            ; the CASE statement avoids the mental conversion to a reduced set of number counts
            ;1 or 2 will be interpreted as '1', 3-5 as '2'; 6 or more as '3'; u as '4'
            case fix(num) of
                0: y(k)= '0'
                1: y(k)= '1'    ;any interpretive program of the result should equate this as 1.5 granules
                2: y(k)= '1'
                3: y(k)= '2'
                4: y(k)= '2'    ;any interpretive program of the result should equate this as 4 granules
                5: y(k)= '2'
                6: y(k)= '3'
                7: y(k)= '3'    ;any interpretive program of the result should equate this as 7.5 granules
                8: y(k)= '3'
                9: y(k)= '4'
            Endcase
            jumpcase:
            print,'file',k,' is in ', y(k),' granules class'
         imout=intarr(3,256,256)       ;conversion to RGB
         for q=0,2 do imout(q,*,*)=imarr(*,*,p)
            outname=strcompress(outdir+sub+'image'+strk+'.jpg',/remove_all)       ;note use of subdirectory "sub",
             write_jpeg,outname,imout,quality=100,/order,/true ;order = 1, (0,0) at top left
            printf,om, k, y(k), format='(I8,A8)'       ;image index and its label, per line of text output
            k=k+1          ;number of sub images advances by 1
        endfor
    endfor          ; splitting of original image concludes
    jumpskipimage:      ; image rejected
endfor           ; goto new image (loop over m index)
read,dummy, prompt='enter to continue to text output'
if dummy ne '' then stop
jumptoout:          ;normal end from within main loop
close,om & free_lun,om   ;the batch output above were replaced by individual output of y(k)
stop
end
```

3.3. Image splitter of sub-images to be classified by models ("predicted").

```
pro image_splitter_5digit_no_label
;takes one 4096 x 4096 tiff image and splits it in 256 (256x256) sub-images
;similar to the previous Utilities. See annotations there.
;for use with ResnNet50, outputs are in "fake RGB", i.e. R, G and B channels have identical content
;no-label version produces sub-images that will be "predicted" by either Locations or Granules program..
dummy='' & inimage='' & side=4096/256 & len=side^2
indir='C:\Users\erios\EMimages_for_prediction\'
outdir='C:\Users\erios\images_for_prediction\here\'
filelist=file_search(indir,'*.tif')
n=n_elements(filelist)
k=0 & nfirst=0 & print,'enter starting value for numbering no-label images, integer:'
read,nfirst,prompt=' starting value for numbering: 0, 256, 512, etc.:'
n_current=nfirst-1  ;thus, if nfirst=0, the generic number k+n_current will start at 0
for l=0,n-1 do begin
    infile=filelist(l)
    img=read_tiff(infile)
    imre=congrid(img,768,768)
    window,0,xsize=768,ysize=768,xpos=0,ypos=0, title=infile
    tv,imre,/order
read,dummy, prompt='enter to continue, c to skip image'
if dummy eq 'c' then goto, jumpskipimage
imarr=bytarr(256,256,len)
k=0        ;k is a counter of subimages, with values between 0 and len-1
n_start=n_current+1        ;because k starts at 0, I add 1 to n_current
    for j=0,side-1 do begin    ;j is row index
       for i=0,side-1 do begin   ;i is column index --horiz. coord.--. rows will be completed first
         imarr(*,*,k)=img(256*i:256*(i+1)-1,256*j:256*(j+1)-1)
         outarr=reform(imarr(*,*,k))
         if RGBswitch eq 1 then begin      ;changes to an RGB format of output
            outarre = intarr(3,256,256)
            for m=0,2 do outarre(m,*,*)=outarr
            outarr = outarre
         endif
           n_current=k+n_start
           strk=strcompress(n_current,/remove_all)      ;will complete numbering length for natural sorting
            ;counter of sub-images in complete set (several EM images)
           if strlen(strk) lt 2 then strk='0000'+ strk
           if strlen(strk) eq 2 then strk = '000'+ strk
           if strlen(strk) eq 3 then strk = '00'+ strk
           if strlen(strk) eq 4 then strk = '0'+ strk
           outname=strcompress(outdir+'image'+strk+'.jpg',/remove_all)
           write_jpeg,outname,outarr,quality=100,/order,/true  ;order = 1, (0,0) at top left
          k=k+1
        endfor
    endfor
jumpskipimage:
;stop
endfor
   print,'last output file number: ',strk
stop
end
```

3.4. Text manipulation utilities

4a.

```
pro correct_label_last_character
; corrects an error, changes integer at end of output line from 9 to 4
; can be easily modified for similar purposes
indir='C:\Users\erios\images_for_training_granules\'     ;change as needed
infile=indir+'Y_list2.txt' & outfile=infile          ;will over-write
origfile=indir+'orig.txt'
file_copy,infile,origfile     ;will use origfile as input
file_delete,infile
infile=origfile          ;these lines prepared a new "Y_list2" file for output
                    ;while preserving the original
openw,om,outfile,/get_lun
openr,im,infile,/get_lun
imdat=''
while ~EOF(im) do begin
    readf,im,imdat,format='(A16)'  ;reads from orig.txt, which must have 16 character lines
                        ;change as needed
if (strmid(imdat,15)) eq '9' then imdat=strmid(imdat,0,15)+'4'
printf,om,imdat,format='(A16)'   ;prints every line to Y_list2.txt
endwhile
close,im & Free_lun,im
close,om & Free_lun,om
stop
end
```

4b.

```
pro add_index_to_labels
; will take a one column list of labels and add an index number to each row
; produces a separate output text document
; first index number selectable as "nfirst"
nfirst= 0 & d=0 & dummy=''
indir='C:\Users\erios\images_3_color_15k_labeled\'
infile=indir+'labels_for_locations.txt'
outdir='C:\Users\erios\images_3_color_no_label\'
outfile=indir+'labels_for_locations_index0.txt'
openw,om,outfile,/get_lun
openr,im,infile,/get_lun
k=nfirst &
while ~EOF(im) do begin
    readf,im,d & print,'d= ',d
    printf,om,strcompress(k,/remove_all),d     ;,format='(2I8)'
    k=k+1
endwhile
print,'k = ',k
close,im & Free_lun,im
close,om & Free_lun,om
stop
end
```

4c.

```
pro squeeze_label_to_range
; changes labels to a narrower range.
outdir='C:\Users\erios\images_for_training_granules\'
outfile=outdir+'Y_list2.txt'
indir='C:\Users\erios\images_for_training_granules\'
infile=indir+'test.txt'
openw,om,outfile,/get_lun
openr,im,infile,/get_lun
imdat=''
while ~EOF(im) do begin
    readf,im,imdat,format='(A16)'
    num=fix(strmid(imdat,15))
    case num of
       0: ad='0'
       2: ad='1'
       4: ad='2'
       7: ad='3'
       9: ad='4'
    ENDCASE
imdat=strmid(imdat,0,15)+ad ; changed character is placed last in line
    printf,om,imdat,format='(A16)'
endwhile
close,im & Free_lun,im
close,om & Free_lun,om
stop
end
```

3.5. Filter for EM images

```
pro EM_image_filter
;image_filter takes tiff images and applies Lee filter algorithm. Outputs to *\filtered
;with 'f' after original name.
dummy=''
b=5  ;spatial frequency parameter for Lee filter
outdir='C:\Users\erios\EM_images_for_granules\filtered\'  ;output directory
indir='C:\Users\erios\EM_images_for_granules\'
inlist=file_search(indir+'*.tif')   ;note, will use all images in folder
n=n_elements(inlist)
for m=0 , n-1 do begin   ;will go through directory and offer to skip file
    infile = inlist(m) & print, 'reading ',infile
    img=float(read_tiff(infile))
    imgr=congrid(img,768,768,cubic=-0.5)
    window,1,xsize=768,ysize=768,xpos=778,ypos=0, title = 'filtered'
    window,0,xsize=768,ysize=768,xpos=0,ypos=0, title = infile
    tvscl,imgr,/order
    ; will apply Lee filter
    imgl=leefilt(img,5)
    imglr=congrid(imgl,768,768,cubic=-0.5)
    wset,1 & tvscl,imglr,/order
    read,dummy, prompt='enter to continue, "c" change, "s" stop : '
    if dummy eq 'c' then goto, jumpskipimage
    if dummy eq 's' then stop
    start=strpos(infile,'\',/reverse_search)+1
    base=strmid(infile,0,start)
    name=strmid(infile,start,26)
    outfile=base+'filtered\'+name+'_f.tif' & print,outfile
    write_tiff,outfile,imgl
    jumpskipimage:        ; image rejected
endfor              ; goto new image (loop over m index)
stop
end
```

3.6. To expand and rotate EM images

```
pro split_15k_rot
;to split EM images obtained at 15k magnification into 4 4096x4096 pixel images
;expanded to an equivalent magnification of 29k,
;offers to perform rotation (to align striations "vertically").
;expands using cubic interpolation, then pads to reach a 8192 by 8192 images
;which are then split into 4 4096 x 4096 EM images suitable for further processing to sub-images
inimage=''
outdir='C:\Users\erios\EM_Images\'
lendir=strlen(outdir)
indir=outdir+'15k\'          ;where source images are placed
filelist=file_search(indir,'*_15k*.tif')  ;uses every tiff file in folder
n=n_elements(filelist)
for k=0,n-1 do begin
    infile=filelist(k)
    common_name=strmid(infile,strpos(infile,'k\')+2)
    first_part=strmid(common_name,0,strpos(common_name,'_15k'))
    outname=outdir+first_part+'_29k'
    img=read_tiff(infile)
    imre=congrid(img,768,768)
    window,0,xsize=768,ysize=768,xpos=0,ypos=0,title=infile
    tv,imre
    print,'option to rotate reference, in degrees, clockwise, always from original'
    an=0 & nxc=100 & anu=0               ;starting angle
jumprotate1:
    read, an, prompt='rotation angle, deg, 50 to continue:'
    if an eq 50 then goto,jumprotate2    ;do not rotate
    anu=an
    imrot=rot((imre),anu,cubic=-0.5)     ;rotation also uses cubic interpolation
    for j=63,767,64 do imrot(*,j)=255    ;a grid for display
    tvscl,(imrot)
    goto,jumprotate1
jumprotate2:
;------------------------------
imrot=rot(img,anu,cubic=-0.5)
expand,imrot,7936,7936,imge    ;imge is original expanded to 29k magnification
 imgex=bytarr(8192,8192)+128     ;to complete array that will be split in 4
 imgex(0,0)=imge          ;fills in the image; last row and last column will be flat grey (value=1
 imgout=bytarr(4,4096,4096) ;buffer for output images
    imgout(0,*,*)=imgex(0:4095,0:4095)
    imgout(1,*,*)=imgex(0:4095,4096:8191)
    imgout(2,*,*)=imgex(4096:8191,0:4095)
    imgout(3,*,*)=imgex(4096:8191,4096:8191)
       for l=0,3 do begin
       outnamel=strcompress(outname+string(l)+'.tif',/remove_all)
       write_tiff,outnamel,imgout(l,*,*)
       endfor
endfor
stop
end
```

Protocol

Preprocessing.

All done with IDL utilities (or similar) provided in Appendix 3.

1. If starting from low magnification (i.e., 15,000) use expander program (Utilities 6), which also allows for rotation in order to align longitudinal striations (myofibrils) with vertical.

2. Use image splitter/classifier, to divide EM images into sub-images and assign a class (label). Work separately for Locations (Utilities 1) and Granules (Utilities 2). These will produce sub-images and a text file of labels.

3. Explore using Lee-filtered images, which may improve visibility of granules and facilitate their counting. Use EM filtering (Utilities 5).
