# Supplementary material for: Artificial intelligence approaches to the volumetric quantification of glycogen granules in EM images of human tissue
Source: J Gen Physiol. 2024 Jul 9;156(9):e202413595. doi: 10.1085/jgp.202413595 (PMC11233403; doi:10.1085/jgp.202413595)
Supplement: Data S2 — shows Python code of the semantic segmentation modules. [file JGP_202413595_DataS2.pdf]

Supplement 2. Python code of the semantic segmentation modules

Includes code for the “Locations” segmenter, a summary of layers for the same, code for the “Granules” segmenter, limited to the core model, as other components are identical to those in “Locations”, and utilities for pre and postprocessing.

Segmenter_Locations_shared. April, 2024.

Cell definitions are dictated by convenience in execution

In [1]:
```
import tensorflow as tf
import numpy as np
from tensorflow import keras
from tensorflow.keras import layers
from tensorflow.keras.layers import Input
from tensorflow.keras.layers import Conv2D
from tensorflow.keras.layers import MaxPooling2D
from tensorflow.keras.layers import Dropout
from tensorflow.keras.layers import Conv2DTranspose
from tensorflow.keras.layers import concatenate
from test_utils import summary   #print(tf.__version__)
import os
import numpy as np # linear algebra
import pandas as pd # data processing, CSV file I/O (e.g. pd.read_csv)
import random
import imageio.v2 as imageio
import matplotlib.pyplot as plt
%matplotlib inline
import sys
import statistics
print(sys.version)  #Python version
```

```
3.11.4 | packaged by conda-forge | (main, Jun 10 2023, 17:59:51) [MSC v.1935 64 bit
(AMD64)]
```

In [2]:
```
path = ''
image_path = os.path.join(path,
         'C:/Users/erios/EM_Images/split_Penn_train/')
mask_path = os.path.join(path,
         'C:/Users/erios/EM_Images/split_Penn_train_locations_labels/')
image_list_orig = os.listdir(image_path)
# list within the folder
mask_list_orig = os.listdir(mask_path)
# list within the folder
image_list = [image_path+i for i in image_list_orig]
# full path and filename
mask_list = [mask_path+i for i in mask_list_orig]
# image_list and mask_list are class lists
image_filenames = tf.constant(image_list)
mask_filenames = tf.constant(mask_list)

dataset = tf.data.Dataset.from_tensor_slices((image_filenames, mask_filenames))
```

Utilities

In [3]:
```
# Preprocess, Data Augmentation
seed = (1, 2)
```

```python
ran = tf.keras.layers.RandomContrast(0.4)
def process_path(image_path, mask_path):
    img = tf.io.read_file(image_path)
    img = tf.image.decode_png(img, channels=1)
    img = tf.image.convert_image_dtype(img, tf.float32)
    mask = tf.io.read_file(mask_path)
    mask = tf.image.decode_png(mask, channels=0, dtype=tf.dtypes.uint16)
    return img, mask

def preprocess(image, mask):
    global seed
    seed = (seed[0] + random.randint(0, 10000), seed[1] + random.randint(0, 10000))
    #changes the seed randomly

    input_image = tf.image.resize(image, (1024,1024), method='nearest')
    input_mask = tf.image.resize(mask, (1024,1024), method='nearest')
    input_image = tf.image.stateless_random_flip_left_right(input_image, seed)
    input_mask = tf.image.stateless_random_flip_left_right(input_mask, seed)
    input_image = tf.image.stateless_random_flip_up_down(input_image, seed)
    input_mask = tf.image.stateless_random_flip_up_down(input_mask, seed)
    input_image = ran(input_image)
    input_image = tf.image.random_brightness(input_image, 0.25)
    return input_image, input_mask

# prediction from model outputs
def create_mask(pred_mask):
    pred_mask = tf.argmax(pred_mask, axis=-1)
    pred_mask = pred_mask[..., tf.newaxis]
    return pred_mask[0]

def show_predictions(dataset=None, num=2):
    """
    Displays the first image of each of the num batches
    """
    if dataset:
        for image, mask in dataset.take(num):
            pred_mask = unet_loc.predict(image)
            display([image[0], mask[0], create_mask(pred_mask)])
    else:
        display([sample_image, sample_mask,
            create_mask(unet_loc.predict(sample_image[tf.newaxis, ...]))])

# utility for display. image tensors must be rank 3
def display(display_list):
    plt.figure(figsize=(15, 15))
    title = ['Input Image', 'True Mask', 'Predicted Mask']
    for i in range(len(display_list)):
        plt.subplot(1, len(display_list), i+1)
        plt.title(title[i])
        plt.imshow(tf.keras.preprocessing.image.array_to_img(display_list[i]))
        plt.axis('off')
    plt.show()
```

Modules

In [4]:
```
def conv_block(inputs=None, n_filters=None, dropout_prob=0, max_pooling=True):
    """
    Convolutional downsampling block
    Arguments:
        inputs -- Input tensor
        n_filters -- Number of filters for the convolutional layers
        dropout_prob -- Dropout probability
        max_pooling -- Use MaxPooling2D to reduce the spatial dimensions
    Returns:
        next_layer, skip_connection --  Next layer and skip connection outputs
    """
    conv = Conv2D(n_filters, 3, activation='relu', padding='same',
                  kernel_initializer='he_normal')(inputs)
    conv = Conv2D(n_filters, 3, activation='relu', padding='same',
                  kernel_initializer='he_normal')(conv)
    if dropout_prob > 0:
        conv = Dropout(dropout_prob)(conv)
    if max_pooling:
        next_layer = MaxPooling2D(2,strides=2)(conv)
    else:
        next_layer = conv
    skip_connection = conv

    return next_layer, skip_connection

def upsampling_block(expansive_input, contractive_input, n_filters=None):
    """
    Convolutional upsampling block
    Returns:
        conv -- Tensor output
    """
    up = Conv2DTranspose(n_filters,3,strides=2,padding='same')(expansive_input)
    merge = concatenate([up, contractive_input], axis=3)
    conv = Conv2D(n_filters, 3, activation='relu', padding='same',
                  kernel_initializer='he_normal')(merge)
    conv = Conv2D(n_filters, 3, activation='relu', padding='same',
                  kernel_initializer='he_normal')(conv)

    return conv

def unet_model7(input_size=(1024, 1024, 1), n_filters=4, n_classes=7):
    """
    Function name changed to unet_model7
    Returns:
    """
    inputs = Input(input_size)
    # Contracting Path (encoding)
    cblock0 = conv_block(inputs, 8)
    cblock1 = conv_block(cblock0[0], 8)
    cblock2 = conv_block(cblock1[0], 16)
    cblock3 = conv_block(cblock2[0], 32)
    cblock4 = conv_block(cblock3[0], 64)
    cblock5 = conv_block(cblock4[0], 128)
    cblock6 = conv_block(cblock5[0], 256, 0.3)
    cblock7 = conv_block(cblock6[0], 512, 0.3, max_pooling=False)
```

```
    # Expanding Path (decoding)
    ublock8 = upsampling_block(cblock7[0], cblock6[1], n_filters * 64)
    ublock9 = upsampling_block(ublock8, cblock5[1],  128)
    ublock10 = upsampling_block(ublock9, cblock4[1],  64)
    ublock11 = upsampling_block(ublock10, cblock3[1],  32)
    ublock12 = upsampling_block(ublock11, cblock2[1],  16)
    ublock13 = upsampling_block(ublock12, cblock1[1],  8)
    ublock14 = upsampling_block(ublock13, cblock0[1],  8)
    conv9 = Conv2D(n_filters, 3, activation='relu', padding='same',
                  kernel_initializer='he_normal')(ublock14)
    conv10 = Conv2D(n_classes, 1, padding='same')(conv9)

    model = tf.keras.Model(inputs=inputs, outputs=conv10)
    return model
```

Model definition, compilation and loading of trained weights

In [ ]:

```
# segmenter location's model is unet_loc
img_height = 1024
img_width = 1024
num_channels = 1

unet_loc = unet_model7((img_height, img_width, num_channels))
# unet_loc.summary()     # generates summary listed below this code
```

In [6]:

```
# uses Nadam optimizer.
# When starting with highly trained weights, use next cell (Adam)
unet_loc.compile(optimizer=tf.keras.optimizers.Nadam(learning_rate=2e-3),    #
              loss=tf.keras.losses.SparseCategoricalCrossentropy(from_logits=True),
    metrics=[tf.keras.metrics.SparseCategoricalAccuracy
             (name= "Sparse Categorical Accuracy")])
```

In [22]:

```
# uses Adam optimizer.
unet_loc.compile(optimizer=tf.keras.optimizers.Adam(learning_rate=5e-4),
              loss=tf.keras.losses.SparseCategoricalCrossentropy(from_logits=True),
              # metrics=['accuracy'])
           metrics=[tf.keras.metrics.SparseCategoricalAccuracy
                   (name= "Sparse Categorical Accuracy")])
```

Model training

In [24]:

```
coarse_tuning_counter = 0
acc = [0.]
loss = [0.]
```

In [ ]:

```
# main training
checkpoint_filepath = 'C:/Users/erios/checkpoints/checkpoint_unet_loc_paul2b'
#2b is for use with 6 labels
```

```
model_checkpoint_callback = tf.keras.callbacks.ModelCheckpoint(
    filepath=checkpoint_filepath,  save_weights_only=True,
                monitor="Sparse Categorical Accuracy",
    mode='max', save_best_only=True)
image_ds = dataset.map(process_path)
processed_image_ds = image_ds.map(preprocess)
#BUFFER_SIZE = 147
#BATCH_SIZE = 9
BUFFER_SIZE = 17
BATCH_SIZE = 3
train_dataset = processed_image_ds.cache().shuffle(BUFFER_SIZE).batch(BATCH_SIZE)
if coarse_tuning_counter == 0:
    initial_epochs = 4          # use in general 5
    total_epochs = initial_epochs
    history = unet_loc.fit(train_dataset, epochs=initial_epochs)
else:
    more_epochs = 20
    total_epochs =  total_epochs + more_epochs
    history = unet_loc.fit(train_dataset, epochs=total_epochs,
        initial_epoch=history.epoch[-1], callbacks=[model_checkpoint_callback])
coarse_tuning_counter += 1
# update history
acc += history.history['Sparse Categorical Accuracy']
loss += history.history['loss']# plot evolution of accuracy and loss
```

In [ ]:

```
# update history
#acc += history.history['Sparse Categorical Accuracy']
#loss += history.history['loss']# plot evolution of accuracy and loss
plt.figure(figsize=(8, 8))
plt.subplot(2, 1, 1)
plt.plot(acc, label='Training Accuracy')
plt.legend(loc='lower right')
plt.ylabel('Sparse Categorical Accuracy')
plt.title('Training Accuracy')
plt.subplot(2, 1, 2)
plt.plot(loss, label='Training Loss')
plt.legend(loc='upper right')
plt.ylabel('Sparse Categorical Cross Entropy')
plt.title('Training Loss')
plt.xlabel('epoch')
plt.ylim([0,2])
plt.show()
```

Prediction on a "validation" or "test" set.

Assumes a ...val_labels folder

In [14]:

```
# this is a "test" dataset --will not be used in training--
path= ''
image_path_val = os.path.join(path,
            'C:/Users/erios/EM_Images/split_randomized_renamed_ping_val/')
mask_path_val = os.path.join(path,
            'C:/Users/erios/EM_Images/split_r_labels_6_val/')
```

```
image_list_orig_val = os.listdir(image_path_val)   # a list of image file names
mask_list_orig_val = os.listdir(mask_path_val)
image_list_val = [image_path_val+i for i in image_list_orig_val]
# a list of image file names that includes the ful path
mask_list_val = [mask_path_val+i for i in mask_list_orig_val]
image_filenames_val = tf.constant(image_list_val)   #tensor, dtype = string
mask_filenames_val = tf.constant(mask_list_val)

dataset_val = tf.data.Dataset.from_tensor_slices
       ((image_filenames_val, mask_filenames_val))
#a dataset of filenames
```

In [15]:

```
# validation .  Preprocess does not include data augmentation
def process_path(image_path, mask_path):
    img = tf.io.read_file(image_path)
    img = tf.image.decode_png(img, channels=1)
    img = tf.image.convert_image_dtype(img, tf.float32)
    mask = tf.io.read_file(mask_path)
    mask = tf.image.decode_png(mask, channels=0, dtype=tf.dtypes.uint16)
    return img, mask

def preprocess_val(image, mask):
    input_image = tf.image.resize(image, (1024,1024), method='nearest')
    input_mask = tf.image.resize(mask, (1024,1024), method='nearest')
    return input_image, input_mask

image_ds_val = dataset_val.map(process_path)
processed_image_ds_val = image_ds_val.map(preprocess_val)
val_dataset = processed_image_ds_val.cache().batch(1)
```

Prediction and output of images as png files

In [ ]:

```
probs_val = unet_loc.predict(val_dataset)   #makes an np probability array  rank 4
n = probs_val.shape[0]                     # the number of images in the val dataset
probs1_val =  np.vsplit(probs_val, n)
#makes a list of n probability arrays (1024, 1024, 2)
pred_m_l_val= [np.zeros((1024,1024,2),dtype=float) for i in range(n)]
#initialization of a list of np.arrays
pred_val_acc_list= [0.0 for i in range(n)]
for i in range (n):
    pred_m_l_val[i] = create_mask((probs1_val[i]))
    #list gets populated by prediction masks
# now will write prediction masks to ping files,
# with same names as original image files in val folder
DIR = "C:/Users/erios/EM_Images/split_r_labels_6_val_preds/"
for i in range(n):
    name=DIR + str(image_list_orig_val[i])
    # list of original image file names
    mask = tf.cast(pred_m_l_val[i],tf.uint16)
    #  had to recast numpy prediction masks as uint16
    pred_png = tf.image.encode_png(mask)
    with open(name, 'wb') as f:
        f.write(pred_png.numpy())                    #  works well
```

Calculates accuracies for the entire val set

and outputs them in a text file

In [17]:
```
m = tf.keras.metrics.Accuracy()
true_m_l_val= [np.zeros((1024,1024,2),dtype=float) for i in range(n)]
#initialization of a list of np.arrays true masks
count = 0
for image, mask in val_dataset:
        true_m_l_val[count] = mask[0]
    # squeezes the first dimension; needed for accuracy calculation
        count=count+1
for i in range(n):
    m.update_state([pred_m_l_val[i]],[ true_m_l_val[i]])
    pred_val_acc_list[i] = m.result().numpy()
    # the accuracy function works in roundabout way
accuracy_out = DIR+"accuracies.txt"
f = open(accuracy_out, "w")
for i in range(n):
    f.write("\n"+str(i)+"    "+str(pred_val_acc_list[i]) )
    # a two-column text output
f.close()
#from statistics import mean
print(pred_val_acc_list,sum(pred_val_acc_list)/14.)
print(statistics.mean(pred_val_acc_list[0:7]),
      statistics.stdev(pred_val_acc_list[0:7]))
# Outputs loss and accuracy lists to text files in DIR.  R
loss_out = DIR+"loss_vs_epoch.txt"       # defines output file name
f = open(loss_out, "w")                  # logical name of output file
for i in range(25):
    f.write("\n"+str(i)+"    "+str(loss[i]) )  # a two-column text output
f.close()
#probs = unet.predict(train_dataset)
#makes an np array (n_elements in train_dataset, 1024, 1024, 2)
probs1 =  np.vsplit(probs, 64)  #makes a list of arrays (1024, 1024, 2)
pred_m_l= [np.zeros((1024,1024,2),dtype=float) for i in range(64)]
#initialization of a list of np.arrays
for i in range (64):
    pred_m_l[i] = create_mask((probs1[i]))      #list gets populated
# The above list can be written to files as with the val set
    # or used for calculation of metrics
```

PRODUCTION

In [ ]:
```
"""a production run requires loading libraries, defining the input dataset,
assembling and compiling the model, defining utilities
(display_prod, create_mask, process_path, preprocess_prod,
show_predictions_prod, and probs_prod, which performs the prediction and outputs th
predicted masks.  # preparing a production dataset.  No masks. """
path= ''
image_path_prod = os.path.join(path,
```

```
                    'C:/Users/erios/EM_Images/037_split_for_segmentation/')
image_list_orig_prod = os.listdir(image_path_prod)   # a list of image file names
image_list_prod = [image_path_prod+i for i in image_list_orig_prod]
# a list of image file names that includes the full path
image_filenames_prod = tf.constant(image_list_prod)   #tensor, dtype = string

dataset_prod = tf.data.Dataset.from_tensor_slices((image_filenames_prod))
#a dataset of filenames
print(len(image_list_prod))
```

In [7]:

```
# prediction from model outputs
def create_mask(pred_mask):
    pred_mask = tf.argmax(pred_mask, axis=-1)
    pred_mask = pred_mask[..., tf.newaxis]
    return pred_mask[0]
    # Production .  Preprocess does not include data augmentation
def process_path(image_path):
    img = tf.io.read_file(image_path)
    img = tf.image.decode_png(img, channels=1)
    img = tf.image.convert_image_dtype(img, tf.float32)

    return img

def preprocess_prod(image):
    input_image = tf.image.resize(image, (1024,1024), method='nearest')
    return input_image

image_ds_prod = dataset_prod.map(process_path)
processed_image_ds_prod = image_ds_prod.map(preprocess_prod)
#prod_dataset is the production dataset, batched and cached
# for application of prediction.
# Not to be confused with dataset_prod
prod_dataset = processed_image_ds_prod.cache().batch(20)
#either way of batching works
```

In [ ]:

```
# Production
probs_prod = unet_loc.predict(prod_dataset)
#  PREDICTS! makes an np probability array  rank 4
n = probs_prod.shape[0]          # the number of images in the prod dataset
probs1_prod =  np.vsplit(probs_prod, n)
#makes a list of n probability arrays (1024, 1024, 2)
pred_m_l_prod= [np.zeros((1024,1024,2),dtype=float) for i in range(n)]
#initialization of a list of np.arrays
pred_prod_acc_list= [0.0 for i in range(n)]
for i in range (n):
    pred_m_l_prod[i] = create_mask((probs1_prod[i]))
    #list gets populated by prediction masks
#display([pred_m_l_prod[4]])   #nice check
# now will write prediction masks to ping files,
# with same names as original image files in val folder
DIR = "C:/Users/erios/EM_Images/037_split_for_segmentation_loc_preds/"
for i in range(n):
    name=DIR + str(image_list_orig_prod[i])
    # list of original image file names
    mask = tf.cast(pred_m_l_prod[i],tf.uint16)
```

```
    #  had to recast numpy prediction masks as uint16 (from int32)
    pred_png = tf.image.encode_png(mask)
    with open(name, 'wb') as f:
        f.write(pred_png.numpy())                    #  works well
```

Studying Metrics

In [ ]:
```
m = tf.keras.metrics.Accuracy()
m.update_state([sample_mask],[ predicted_mask])
m.result().numpy()
```

In [ ]:
```
mi = tf.keras.metrics.IoU(num_classes=2, target_class_ids=[1])
mi.update_state([sample_mask],[ predicted_mask])
mi.result().numpy()
```

In [ ]:
```
mi = tf.keras.metrics.IoU(num_classes=2, target_class_ids=[0])
mi.update_state([sample_mask],[ predicted_mask])
mi.result().numpy()
```

Summary of Layers.

Layer (type)	Output Shape	Param #	Connected to
input_1 (InputLayer)	[(None, 1024, 1024, 1)]	0	[]
conv2d (Conv2D)	(None, 1024, 1024, 8)	80	['input_1[0][0]']
conv2d_1 (Conv2D)	(None, 1024, 1024, 8)	584	['conv2d[0][0]']
max_pooling2d (MaxPooling2D)	(None, 512, 512, 8)	0	['conv2d_1[0][0]']
conv2d_2 (Conv2D)	(None, 512, 512, 8)	584	['max_pooling2d[0][0]']
conv2d_3 (Conv2D)	(None, 512, 512, 8)	584	['conv2d_2[0][0]']
max_pooling2d_1 (MaxPooling2D)	(None, 256, 256, 8)	0	['conv2d_3[0][0]']
conv2d_4 (Conv2D)	(None, 256, 256, 16)	1168	['max_pooling2d_1[0][0]']
conv2d_5 (Conv2D)	(None, 256, 256, 16)	2320	['conv2d_4[0][0]']
max_pooling2d_2 (MaxPooling2D)	(None, 128, 128, 16)	0	['conv2d_5[0][0]']
conv2d_6 (Conv2D)	(None, 128, 128, 32)	4640	['max_pooling2d_2[0][0]']
conv2d_7 (Conv2D)	(None, 128, 128, 32)	9248	['conv2d_6[0][0]']
max_pooling2d_3 (MaxPooling2D)	(None, 64, 64, 32)	0	['conv2d_7[0][0]']
conv2d_8 (Conv2D)	(None, 64, 64, 64)	18496	['max_pooling2d_3[0][0]']
conv2d_9 (Conv2D)	(None, 64, 64, 64)	36928	['conv2d_8[0][0]']
max_pooling2d_4 (MaxPooling2D)	(None, 32, 32, 64)	0	['conv2d_9[0][0]']
conv2d_10 (Conv2D)	(None, 32, 32, 128)	73856	['max_pooling2d_4[0][0]']
conv2d_11 (Conv2D)	(None, 32, 32, 128)	147584	['conv2d_10[0][0]']
max_pooling2d_5 (MaxPooling2D)	(None, 16, 16, 128)	0	['conv2d_11[0][0]']
conv2d_12 (Conv2D)	(None, 16, 16, 256)	295168	['max_pooling2d_5[0][0]']
conv2d_13 (Conv2D)	(None, 16, 16, 256)	590080	['conv2d_12[0][0]']
dropout (Dropout)	(None, 16, 16, 256)	0	['conv2d_13[0][0]']
max_pooling2d_6 (MaxPooling2D)	(None, 8, 8, 256)	0	['dropout[0][0]']
conv2d_14 (Conv2D)	(None, 8, 8, 512)	1180160	['max_pooling2d_6[0][0]']
conv2d_15 (Conv2D)	(None, 8, 8, 512)	2359808	['conv2d_14[0][0]']
dropout_1 (Dropout)	(None, 8, 8, 512)	0	['conv2d_15[0][0]']
conv2d_transpose (Conv2DTranspose)	(None, 16, 16, 256)	1179904	['dropout_1[0][0]']
concatenate (Concatenate)	(None, 16, 16, 512)	0	['conv2d_transpose[0][0]', dropout[0][0]']
conv2d_16 (Conv2D)	(None, 16, 16, 256)	1179904	['concatenate[0][0]']
conv2d_17 (Conv2D)	(None, 16, 16, 256)	590080	['conv2d_16[0][0]']
conv2d_transpose_1 (Conv2DTranspose	(None, 32, 32, 128)	295040	['conv2d_17[0][0]']
concatenate_1 (Concatenate)	(None, 32, 32, 256)	0	['conv2d_transpose_1[0][0]', 'conv2d_11[0][0]']
conv2d_18 (Conv2D)	(None, 32, 32, 128)	295040	['concatenate_1[0][0]']
conv2d_19 (Conv2D)	(None, 32, 32, 128)	147584	['conv2d_18[0][0]']
conv2d_transpose_2 (Conv2DTranspose	(None, 64, 64, 64)	73792	['conv2d_19[0][0]']
concatenate_2 (Concatenate)	(None, 64, 64, 128)	0	['conv2d_transpose_2[0][0]', 'conv2d_9[0][0]']
conv2d_20 (Conv2D)	(None, 64, 64, 64)	73792	['concatenate_2[0][0]']
conv2d_21 (Conv2D)	(None, 64, 64, 64)	36928	['conv2d_20[0][0]']
conv2d_transpose_3 (Conv2D)	(None, 128, 128, 32)	18464	['conv2d_21[0][0]']
concatenate_3 (Concatenate)	(None, 128, 128, 64)	0	['conv2d_transpose_3[0][0]', 'conv2d_7[0][0]']

conv2d_22 (Conv2D)	(None, 128, 128, 32)	18464	['concatenate_3[0][0]']
conv2d_23 (Conv2D)	(None, 128, 128, 32)	9248	['conv2d_22[0][0]']
conv2d_transpose_4 (Conv2DTranspose)	(None, 256, 256, 16)	4624	['conv2d_23[0][0]']
concatenate_4 (Concatenate)	(None, 256, 256, 32)	0	['conv2d_transpose_4[0][0]', 'conv2d_5[0][0]']
conv2d_24 (Conv2D)	(None, 256, 256, 16)	4624	['concatenate_4[0][0]']
conv2d_25 (Conv2D)	(None, 256, 256, 16)	2320	['conv2d_24[0][0]']
conv2d_transpose_5 (Conv2D)	(None, 512, 512, 8)	1160	['conv2d_25[0][0]']
concatenate_5 (Concatenate)	(None, 512, 512, 16)	0	['conv2d_transpose_5[0][0]', 'conv2d_3[0][0]']
conv2d_26 (Conv2D)	(None, 512, 512, 8)	1160	['concatenate_5[0][0]']
conv2d_27 (Conv2D)	(None, 512, 512, 8)	584	['conv2d_26[0][0]']
conv2d_transpose_6 (Conv2DTranspose)	(None, 1024, 1024, 8)	584	['conv2d_27[0][0]']
concatenate_6 (Concatenate)	(None, 1024, 1024, 16)	0	['conv2d_transpose_6[0][0]', 'conv2d_1[0][0]']
conv2d_28 (Conv2D)	(None, 1024, 1024, 8)	1160	['concatenate_6[0][0]']
conv2d_29 (Conv2D)	(None, 1024, 1024, 8)	584	['conv2d_28[0][0]']
conv2d_30 (Conv2D)	(None, 1024, 1024, 4)	292	['conv2d_29[0][0]']
conv2d_31 (Conv2D)	(None, 1024, 1024, 7)	35	['conv2d_30[0][0]']

==
Total params: 8656655 (33.02 MB)
Trainable params: 8656655 (33.02 MB)
Non-trainable params: 0 (0.00 Byte)

Segmenter_Granules. Core model definition only. April 17, 2024.

In [19]:
```
def unet_model(input_size=(1024, 1024, 1), n_filters=4, n_classes=2):
    """
    Returns:
        model -- tf.keras.Model
    """
    inputs = Input(input_size)
    # Contracting Path (encoding)
    cblock0 = conv_block(inputs, 4)
    cblock1 = conv_block(cblock0[0], 8)
    cblock2 = conv_block(cblock1[0], 16)
    cblock3 = conv_block(cblock2[0], 32)
    cblock4 = conv_block(cblock3[0], 64)
    cblock5 = conv_block(cblock4[0], 128)
    cblock6 = conv_block(cblock5[0], 256, 0.3) # Include a dropout_prob of 0.3 for
    cblock7 = conv_block(cblock6[0], 512, 0.3, max_pooling=False)
    # Expanding Path (decoding)
    ublock8 = upsampling_block(cblock7[0], cblock6[1], n_filters * 64)
    ublock9 = upsampling_block(ublock8, cblock5[1],  128)
    ublock10 = upsampling_block(ublock9, cblock4[1],  64)
    ublock11 = upsampling_block(ublock10, cblock3[1],  32)
    ublock12 = upsampling_block(ublock11, cblock2[1],  16)
    ublock13 = upsampling_block(ublock12, cblock1[1],  8)
    ublock14 = upsampling_block(ublock13, cblock0[1],  n_filters)
    conv9 = Conv2D(n_filters, 3, activation='relu', padding='same', kernel_initiali
    conv10 = Conv2D(n_classes, 1, padding='same')(conv9)

    model = tf.keras.Model(inputs=inputs, outputs=conv10)
    return model
```

In [ ]:

Utilities.

Ancillary programs that implement and streamline pre & postprocessing of EM images. All are written in IDL language (Harris Geospatiale, Paris, France), a stable, well-supported programming environment with Fortran-like syntax. Programs are commented (green font) for easy transcription to other languages, including Python. Python has modules for automatic conversion of IDL script. Additional utilities are shared with the Categorical Classifiers, in Supplement 1. Additional guidance in the use of these utilities is available upon request to the corresponding author.

Granule_density. The utility that takes the outputs of the two segmenters to calculate fraction of pixels *pf*

```
pro granule_density
;reads output from semantic segmenters "Locations" and "Granules" and calculates pixel fraction in granules, at every location
;note, no output for unclassified areas
;note, Z and mitochondria included in saame location label
;note, "region" and "location" are used indistinctly
;note, "density" and "fraction" are used indistinctly
outdir='C:\Users\erios\EM_Images\'
indir1='C:\Users\erios\EM_Images\037_split_for_segmentation_loc_preds\'          ; totals pixels in different locations (area of regions
indir2='C:\Users\erios\EM_Images\037_split_for_segmentation_granules_preds\'     ; totals pixels in granules, at different locations
filelist1=file_search(indir1,'*.png')                                 ; finds location prediction files in folder
filelist2=file_search(indir2,'*.png')                                 ; finds granules prediction files in folder
n=n_elements(filelist1)
if n ne n_elements( filelist2) then begin
    print, 'unequal numbers in input files' & stop
endif
foutname1=outdir+'location and granule areas Montse Penn.txt'              ; defines text output files
foutname2=outdir+'granule densities Montse Penn.txt'

openw,om1,foutname1,/get_lun
openw,om2,foutname2,/get_lun
printf,om1, 'image_name                           A_area A_grns I_area I_grns Z_area Z_grns S_area S_grns N_area N_grns'
printf,om2, 'image_name                           A_dens   I_dens   Z_dens   S_dens   N_dens'
for i = 0, n-1 do begin
    imloc = read_png(filelist1(i))
    imgra = read_png(filelist2(i))
    fullname = filelist1(i)
    name = strmid(fullname,strpos(fullname,'\', /Reverse_search)+1)
    short_name = strmid(name,0,35)
    vec1 = lonarr(10) & vec2 = fltarr(5)
    for j = 1,5 do begin                                           ; loop over locations
        roij = where (imloc eq j,count)
        if count ne 0 then begin
           locj = n_elements(roij)                                  ; number of pixels at location j
           granulej = total(imgra(roij))                               ; number of pixels in granules at location j
           densj = float(granulej)/locj                               ; fraction of pixels in granules
           endif else begin
             locj = 0
             granulej = 0
             densj = -1          ;not defined
        endelse
;preparation of output lists
        vec1(2*(j-1))=locj                                          ; number of pixels in region j
        vec1(2*j-1) = granulej
        vec2(j-1) = densj
    endfor
    printf,om1,short_name,vec1, format='(A35,I8,9I7)'
    printf,om2,short_name,vec2, format='(A35,5F8.4)'
;stop ; for debugging
endfor
close,om1 & free_lun,om1
close,om2 & free_lun,om2
stop
```

Split_EM_to_Newdim.   A preprocessing utility that splits large EM images to the input shape of segmenters.

```
pro split_EM_to_newdim_png
;to split large images, usually 4096x4096, to input size for segmenters (1024 x 1024, 0.8 nm/pixel)
;may include rotation of images
;input files sub-folder named by editing
;version is good for all magnifications
inimage=''
outdir='C:\Users\erios\EM_Images\split_for_segmentation\'       ;set input folder
indir='C:\Users\erios\EMimages_for_prediction_September22\'
lendir=strlen(outdir)
filelist=file_search(indir,'*.tif')
n=n_elements(filelist)
dummy=''
split=4                                                         ;number of split segments in one dimension.
                                                                ;valid for 15 k magnification.
sub=split^2                                                     ;Number of sub-images will be split^2
newdim=4096/split                                               ;assumes input images are 4096x4096 pixels
for k=0,n-1 do begin
    infile=filelist(k)
    common_name=strmid(infile,strpos(infile,'11\')+3,25)        ;adapt positional marker in string to input folder
    outnam = outdir+common_name+'_'
    img=read_tiff(infile)
    if n_elements(img(0,*)) ne 4096 then goto,jumpskipimage
    imre=congrid(img,768,768)
    window,0,xsize=768,ysize=768,xpos=0,ypos=0,title=infile
    tv,imre
    read,dummy, prompt = 'any to skip, enter to continue;'
    if dummy ne '' then goto,jumpskipimage
;     goto,jumprotate4                                          ;skips rotation option
 jumprotate4:                                                   ;skips rotation stage
    print,'starting on '+infile
    imgout=bytarr(newdim,newdim)                                ;buffer for output images
    l=0
    for i=0,4096-split,newdim do begin
       for j=0,4096-split,newdim do begin
       imgout = img(i:i+newdim-1,j:j+newdim-1)
       outname=strcompress(outnam+string(l)+'.png',/remove_all)
        write_png,outname,imgout
        print,'output '+ outname
        l=l+1
        endfor
    endfor
    jumpskipimage:
;stop   ; debugging
endfor
stop
end
```
