# Supplementary material for: Artificial intelligence approaches to the volumetric quantification of glycogen granules in EM images of human tissue
Source: J Gen Physiol. 2024 Jul 9;156(9):e202413595. doi: 10.1085/jgp.202413595 (PMC11233403; doi:10.1085/jgp.202413595)
Supplement: Data S3 — shows simulator of granules in a volume and detector and measurer of granules in label masks. [file JGP_202413595_DataS3.pdf]

Supplement 3. Simulator of granules in a volume and detector and measurer of granules in label masks.

Programs written in IDL language (Harris Geospatiale, Paris, France).

```
pro voxel1
; Fills a volume of dimensions side, side, sidez with an arbitrary number "ngrans"
; of granules of random radius ("randomn", normal distribution)
; and random ("randomu", uniform distribution) center locations x,y,z
; outputs the list of granule centers and radii (array gsites) in text format.
; outputs the volumes at chosen numbers of granules as unformatted files, extension .u.
; may output as tiff files (400 "channels", 400 x 200 "images").
; still shows a few errors in output (high values of vol outside granules)
; which require use of restore_vol_granules.pro for cleaning..
dir='D:\Dropbox\Methods Manuscript Revision\'        ;defines folder for output
side=400 & sidez=200                                 ; numbers of voxels are scaled by 0.4. Prism has square base, .
; It scales down a 0.5 cubic micron volume.
ngrans = 3000 & ncount = 0   ; desired maximum number of granules and granule counter.
; Corresponds to a maximum density of 6000 grans/cubic micron
vol = bytarr(side, side, sidez)                      ; where granules will be deposited
roi = vol                                            ; mirror volume where voxels of new granule accumulate temporarily
; the following 5 just read an unformatted (saved) volume >>>>>>>>>>>>>>REMOVE BETWEEN GREEN >>> IN NORMAL USE
;   inname = dir +'vol_400_1600grans.u'
;   openr,um,inname,/get_lun & readu, um, vol & close,um & free_lun,um & ncount=1600
;>>>>>>>>>>>>>REMOVE BETWEEN GREEN IN NORMAL USE >>>>>>>>>>>>>>>>>>>>>>>>>>>>>>>>>>>>>>>>>>>>>>>>>>>>>>>>>>>>>>
outname= dir + 'gran_centers_radii_'+strcompress(side,/remove_all)+'_'+strcompress(ngrans,/remove_all)+'.txt'
; will output list of granule centers & radii
openw,om,outname,/get_lun                            ; just opens a file for output of list
s = size(vol)                                        ; a vector with dimensions of volume
scale3, XRANGE=[0, S[1]], YRANGE=[0, S[2]], ZRANGE=[0, S[3]]  ; invokes subroutine that enables later display of volume
sm = side - 1 & szm = sidez - 1                      ; for ease of programming
gsites = fltarr (ngrans, 6)                          ; lists granules, with index, location x y z and radius
; x, y, z in (i,1), (i,2) and (i,3) respectively. radius in (i, 4), number of voxels in (i,5).
while ncount lt ngrans do begin                      ; big loop on granules; will first find radius
    r = 5.0 + 1.0 * randomn(seed)                    ; checked distribution, scaled 2.5 down
    r=round(r)                                       ; rounds to integer number
    if r lt 2 then r = 2                             ; minimum radius, corresponds to 10 microns diameter
    if r gt 9 then r =9                              ; maximum radius, corresponds to 45 microns
    nel=[27,93,251,484,895,1365,2100,2900,4000]      ; reduced
    nelements=nel(r-2)                               ; nelements will be used to check granule completion
    nel_red = [23,80,200,430,750,1250,1950,2750,3600]  ;to remove grossly incompletes
    nelements_red=nel_red(r-2)
    x = r + round(randomu(seed)*(side-2*r))          ; x location of granule, avoids borders
    y = r + round(randomu(seed)*(side-2*r))
    z = r + round(randomu(seed)*(sidez-2*r))
    ;THE CORE OF THE PROGRAM
    ;will go over coordinates of volume deciding whether it is within the granule of radius r centered at x,y,z
    voxelcount = 0
    for j=0,sm do begin                              ; check if voxel is within granule. loop on j, abscissa of volume.
       for k=0,sm do begin                           ; loop on k, ordinate of volume
          for l=0,szm do begin                       ; loop on vertical axis of volume
          ; start voxel assignment
          if ((x-j)^2+(y-k)^2+(z-l)^2) lt r^2 then begin   ;voxel j,k,l of volume is within granule
              if vol(j,k,l) eq 255 then begin        ; but it could overlap other granules
                  goto,jump_discard                  ; skip the new granule and skip update of number when new gr overlaps
              endif
          roi(j,k,l) = 255                           ;accepts voxel in temp granule located in volume roi, mirroring vol
          voxelcount = voxelcount + 1
           if voxelcount eq nelements then begin
              goto,jump_gran_completed               ; expected to save time.
          endif
        endif                                        ; end of tasks to do when voxel is in granule
        endfor                                       ; ends check on  volume coordinate l
      endfor                                         ; ends check on  volume coordinate k
   endfor                                            ; ends check on  volume coordinate j
   jump_gran_completed:
   vol(where(roi eq 255)) = 255                      ;the new granule is placed into vol
   wait,0.1
print,'granule ' + strcompress(ncount), ',  x y z =', x, y, z, ',   r =',strcompress(r), $
', nvoxels =',strcompress(voxelcount)
; new granule has been approved and placed in volume.  Now must do some housekeeping
   roi=bytarr(side,side,sidez)                       ; roi must be reset to zeros
   gsites(ncount,*) = [ncount,x,y,z,r,voxelcount]    ; granule parameters stored in array gsites(i,*)
   printf,om,gsites(ncount,*),format='(6I7)'         ; this is the actual write to output command
   if ncount Mod 400 eq 0 then begin                 ; will save vol at 400 granules and multiples of 400
      nameu= dir + 'vol_'+strcompress(side,/remove_all)+'_'+strcompress(ncount,/remove_all)+'grans.u'
      openw,um,nameu,/get_lun                        ; an empty output file must be opened for writing
      writeu,um,vol                                  ; the actual output (saving) command
      close,um & free_lun,um                         ; the output file must be closed
   endif                                             ; ends the output of intermediate density volume
   ncount = ncount + 1
jump_discard:                                        ; skip the new granule and skip update when new gr overlaps
endwhile
close,om & free_lun, om                              ; closes the text file of granule centers and radii
thresh=254 & shade_volume, vol, thresh, v,p          ; high-level subroutine to generate 3D rendering
window,0, xsize=700, ysize=500 & tv,polyshade(v,p,/T3D)     ; defines a window and displays the volume
;with the full count of granules
window,1, xpos = 0, ypos=0, xsize=side, ysize=side & tv,vol(*,*,sidez/2)    ; displays the central x-y plane
nameu= dir + 'vol_'+strcompress(side,/remove_all)+'_'+strcompress(ngrans,/remove_all)+'grans.u'
openw,um,nameu,/get_lun                              ; outputs the last volume in "unformatted" format
writeu,um,vol                                        ; with name "nameu", extension .u
close,um & free_lun,um                               ; closes output file
;optional tiff output
;namet= dir + 'vol_'+strcompress(side,/remove_all)+'_'+strcompress(ngrans,/remove_all)+'grans.tif'
;write_tiff,namet,vol
stop
end
```

A module that corrects minor errors in the volumes generated by voxel1.pro.

```
pro restore_vol_granules
; corrects minor errors in volume produced by voxel1
side=400 & sidez=200 & ngrans=3000
line = fltarr(6) & gsites=fltarr(ngrans,6)
outname='D:\Dropbox\Methods Manuscript Revision\gran_centers_radii_400_3000.txt'
dir='D:\Dropbox\Methods Manuscript Revision\'
side=400 & sidez=200 & ngrans=3000
openr,om,outname,/get_lun                          ; first load centers and radii produced by voxel1
for i=0,ngrans-1 do begin
    readf,om,line
    gsites(i,*)=line
endfor
close,om & free_lun,om
;actual restoration of granules in volume vil
vil=bytarr(400,400,200)
s = size(vil)
scale3, XRANGE=[0, S[1]], YRANGE=[0, S[2]], ZRANGE=[0, S[3]]   ;scale3 module,enables later display of volume
for i=0,2999 do begin
    x=gsites(i,1) & y=gsites(i,2) & z=gsites(i,3) & r=gsites(i,4)
    nel=[27,93,251,484,895,1365,2100,2900,4000]
    nelements=nel(r-2)                             ; nelements will be used to check granule completion
    voxelcount=0
    for j=0,399 do begin
        for k=0,399 do begin
            for l=0,199 do begin
                if ((x-j)^2 + (y-k)^2 +(z-l)^2) lt r^2 then begin
                    vil(j,k,l)=255
                    voxelcount = voxelcount + 1
                endif
                if voxelcount eq nelements then goto,jump_gran_completed
            endfor
        endfor
    endfor
    jump_gran_completed:
    wait, 0.1
    print,'restored granule ',i
    if ((i Mod 400 eq 0) and (i ne 0)) then begin   ; will save vol at grans count 400 and multiples
        nameu= dir + 'vil_'+strcompress(s(1),/remove_all)+'_'+strcompress(i,/remove_all)+'grans.u'
        openw,um,nameu,/get_lun
        writeu,um,vil
        close,um & free_lun,um
    endif
endfor
thresh=254 & shade_volume, vil, thresh, v, p
window,0, xsize=700, ysize=400 & tv,polyshade(v,p,/T3D)
window,1, xpos = 0, ypos=0, xsize=side, ysize=side & tv,vil(*,*,100)
nameu= dir + 'vil_'+strcompress(s(1),/remove_all)+'_'+strcompress(i,/remove_all)+'grans.u'
openw,um,nameu,/get_lun
writeu,um,vil            ;saves the  volume with maximal count of granules.
close,um & free_lun,um
stop
end
```

Program to detect and measure granules in label masks. For convenience of presentation, the code is displayed as 3 separate images.

```
pro g2_xy_granules_full_folder
; produces list of granule parameters, from label images
; assumes pixel distances of 0.8 nm, but all that can be changed
; all png files in folder are processed.
; All granule parameters are output on same line, with number of image and granule
; this program also produces images with event contour and long axis,
; see Brum et al. J. Physiol. 2000, for examples
; it uses as input label files .png..
; adapted to detect segmentation labels of granules, has very simple criteria for event detection

device, get_decomposed=olddc      ; to work with TRUE color
device,decomposed=0
dummy=''
storage= 'C:\Users\erios\EM_Images\split_granules_Montse_Penn_train_labels\'
;where input label files will be located.  May change if necessary.
dir1= 'C:\Users\erios\EM_Images\split_granules_Montse_Penn_train\'       ;where input files will be located.
dir='D:\Dropbox\Methods Manuscript Revision\'
table=3; contrast=0.0
xlen=1024 & xfst=1 & nx=1024        ; file dimensions, change if necessary.
ylen=1024 & ny=1024
    loadct,table &tvlct,r,g,b,/get&r(0)=0 & g(0)=255
    r(221)=255& g(221)=0 & b (221)=0
    r(220)=0 & g(220)=0 & b(220)=0
    r(219)=0 & g(219)=0 & b(219)=255
    window,3,xs=xlen/2,ys=ylen/2,xpos=0,ypos=0
dummy='' & fnama='' & dummys='' & outnama=''
dx = 0.8 & dy = dx & tscan =dy & dt =dx
cri=1.
cris=200
;input file block ********************************************
unity=fltarr(xlen,ylen)+1.
m_pixel=50                        ;minimum pixel size, a criterion for exclusion
filelist=file_search(storage,'*.png')
n=n_elements(filelist)
print,'n = ', n
tcount=0                          ;total counter of events
pro_sk=(fltarr(50000.,9))               ;for output list
for k=0,n-1 do begin                   ;loop over images
print, 'k =',strcompress(k)
    infile=filelist((k))
    ima=read_png(infile) & ima=long(ima)
    imac=rebin2(ima)
    npx=xlen & ny=ylen & npy=ny & wid=npy & init=0 & fin=xlen-1
    xmin=0 & xmax=xlen-1 & nl=0 & nnl=ylen-1 & area=npx*npy*dx*dx
    ;true area, even though calculated in compressed image
    yf2=ima
    jump_defmask:
    mask=ima                      ;el    iminates all prior filtering
    loadct,table
    im= ima
    siim=1010
    im(siim:nx-1,siim:ny-1)=0             ; no starting beyond 1010
    ime=ima
    mask=ima                      ;Array to hold final event masks
    imo=ima*0+220                   ;imo will be used to display event contours and axes
```

Continues on next page

```
d=ima(where (im eq 0))                ;(imf eq 0))
mean=0
sq=fltarr(1)
skc=0                           ;initalize event counter
While (total(im) ne 0) do begin
   ;print,'total im = ',total(im)
   abt=min(where(im eq 1)) & tt=abt/npx & xx=abt mod npx ;initial position of event region
   ;define search area
   nnk=fix(min([37, xx]))    ;starts 4 microns from edge
   if npx-xx lt 37 then nnk=73-(npx-xx)
   nnr=fix(max([72-xx, 72-nnk]))  ;i changed from 0 to 2 the condition
   mmb=fix(min([37, tt]))
   if npy-tt lt 37 then mmb=73-(npy-tt)
   mme=fix(max([72-tt, 72-mmb]))
   ym=lonarr(mmb+mme+1,mmb+mme+1)     ;array to hold growing points
   ym(36,36)=1                  ;initial seeding for growth
   sk=ym                       ;array to hold  the spark as seen in the mask image
   for iii=0,500 do begin               ;surface growth generation count
     yt=sk                     ;potential new surface points
     nend=1
     jump_enlarge:
     nend=nend+1
     yn=(ime(xx-nnk:xx+nnr,tt-mmb:tt+mme)) and long(smooth(double(ym),3,edge=1)*100.<1)
                    ;dilation of ym by 3*3 filter
     if total(yn) eq 0 and iii eq 0 then begin
      for w=0,nend do for v=0,nend do ym(0 > nnk+w < 72,0 > mmb+v < 72)=1
      goto, jump_enlarge
     endif
     if total (yn) eq 0 then goto, jump2
                    ;no furth growth, stop and update spark count
     sk=sk>yn                 ;update sk
     ime(xx-nnk:xx+nnr,tt-mmb:(tt+mme))=ime(xx-nnk:xx+nnr,tt-mmb:(tt+mme))-fix(sk)>0
                    ;excise the points that already included in the cluster
     im(xx-nnk:xx+nnr,tt-mmb:(tt+mme))=im(xx-nnk:xx+nnr,tt-mmb:(tt+mme))-fix(sk)>0
     ym=yn-long(yt)>0           ;true new surface growth point
     if iii eq 500 then print,'WARNING: event SEARCH AREA MAY BE TOO SMALL'
   endfor
   jump2: sd=sd
   s_mass=total((ima(xx-nnk:xx+nnr,tt-mmb:(tt+mme)<siim-1)-mean)*sk) ; calculation of signal mass
   sizsk=size(sk)
   sq= s_mass/(sd*sqrt(total(sk)))
   if  total(sk) eq 1 then begin
     im(xx,tt)=0
         mask(xx,tt)=0
         goto, jump3
   endif
   if  total(sk) le m_pixel then mask(xx-nnk:xx+nnr,tt-mmb:(tt+mme)$
   <siim-1)=mask(xx-nnk:xx+nnr,tt-mmb:(tt+mme)<siim-1)*(1-sk) ; remove the unlikely events in the mask
   if  total(sk) le m_pixel then goto, jump3 ;discard if the false rate > 0.1 per 768*206 image
  if  s_mass/total(sk) le cri*m_pixel/cris then begin
   mask(xx-nnk:xx+nnr,tt-mmb:(tt+mme)<siim-1)=mask(xx-nnk:xx+nnr,tt-mmb:(tt+mme)<siim-1)*(1-sk)
   ; remove the unlikely events in the mask
   goto, jump3
  endif
   ars=rebin(float(sk),nnk+nnr+1,1) & ars=where(ars ne 0)
```

Continues on next page

```
        ax=max(ars) & bx=min(ars)
        ars=rebin(float(sk),1,(size(sk))(2)) & ars=where(ars ne 0)
        at=max(ars) & bt=min(ars)
        ux=xx-nnk & ut=tt-mmb
        bb=size(sk) & c=size(yf2) & nppx=c(1)
        am=rebin(float(sk),bb(1),1) & am=where(am ne 0)
        ax=max(am) & bx=min(am)                 ; ax,bx: left and right edges of event within mask
        widthx=dx*(ax-bx)
        am=rebin(float(sk),1,bb(2)) & am=where(am ne 0)
        at=max(am) & bt=min(am)                 ; at,bt: initiation and end time of event within mask
        widthy=dx*(at-bt)
        areask=dx*dx*n_elements(where(sk gt 0))
        cim=ima(ux:ux+bb(1)-1,ut:ut+bb(2)-1) ; cim is filtered image within mask
        tim=yf2(ux:ux+bb(1)-1,ut:ut+bb(2)-1) ; tim, normalized image within mask
        skc=skc + 1                     ; event counter incremented
        tcount=tcount + 1 & if tcount Mod 50 eq 0 then print, 'spark total #:',tcount
        ;work with contour
        sk1=sk*0 & sk1(*,0)=1 & sk1(bb(1)-1,*)=1 & sk1(*,bb(2)-1)=1 & sk1(0,*)=1
        ss=(shift(sk,1,0)+shift(sk,-1,0)+shift(sk,0,1)+shift(sk,0,-1))<1-sk
        border=where(ss ne 0)
        posi=bytarr(n_elements(border),2)
        np=n_elements(border)
        for i=0,np-1 do posi(i,0)=border(i) Mod bb(1) ;x array index within sk
        for i=0,np-1 do posi(i,1)=border(i)/ bb(1)    ;y array index within sk
        minx=min(posi(*,0)) & maxx=max(posi(*,0)) & mint=min(posi(*,1)) & maxt=max(posi(*,1))
        pos=posi*dx
        dis=fltarr(np,np)
       for i=0,np-1 do for jj=0,np-1 do dis(i,jj)=sqrt((pos(i,0)-pos(jj,0))^2+(pos(i,1)-pos(jj,1))^2)
        length=max(dis)
        whemax=where(dis eq length)
;      stop
        imax=whemax mod np & ifst=imax(0)<imax(n_elements(imax)-1) & ilst =imax(0)>imax(n_elements(imax)-1)
        gx= ux+ total(cim(where(sk ne 0)) * (where(sk ne 0) mod bb(1)))/total(cim(where(sk ne 0)))
        gy= ut+ total(cim(where(sk ne 0)) * ((where(sk ne 0)/bb(1))))/total(cim(where(sk ne 0)))
        ;center of gravity within ROI
        whemax=where(tim*sk eq max(tim*sk)) & whemax=whemax(0)
        maxsk=fix(whemax/bb(1))             ; t position of maximum within mask
        mask2=mask
        for w=-3,3 do for v=-3,3 do mask2(0 > gx+w < 1023,0 > gy+v < 1023 )=-1
        tvscl,rebin(mask2,512,512)
        ;stop
        pro_sk(tcount-1,0)=skc              ; event number
        pro_sk(tcount-1,1)=round(gx)
        pro_sk(tcount-1,2)=round(gy)    ; center of gravity within ROI
        pro_sk(tcount-1,3)=length
        pro_sk(tcount-1,4)=widthx
        pro_sk(tcount-1,5)=widthy
        pro_sk(tcount-1,6)=areask
        pro_sk(tcount-1,7)=k
        pro_sk(tcount-1,8)=tcount
        jump3:  sd=sd
    endwhile
    jump5: ;orderly exit
    if skc le 0 then goto,jump4
    jump4: sd=sd   ;go to another image
    cnt=(shift(mask,-1,0)+shift(mask,1,0)+shift(mask,0,1)+shift(mask,0,-1))<1-mask>0 ;contour of all sparks
    cntc=rebin(cnt*10,xlen/2,ylen/2)
    tv,100+cntc*200<255                    ; contour  of all events
    ;stop  ;use the following to display original image
    ;fileimage= dir1+strmid(filelist(k),65,4) +'.png' & image= read_png(fileimage)
    ;tvscl,rebin2(image)  ;tvscl,rebin2(mask2)
    jump04:
endfor
pro_sk=pro_sk(0:tcount-1,*)
    tava=transpose(pro_sk)
    outnamo = strmid(filelist(k-1),0,74)+'_all.txt'     ;  ascii output with event parameters
    openw,un,outnamo,/get_lun
    printf,un,'gran #   gx      gy     length width x width y  n_pixel image#'
    printf,un,format='(3I8,4f8.1,2I8)',tava

    close,un & free_lun,un
stop
device, decomposed=olddc
END
```
